# Beyond Packaging: A Perspective on the Emerging Applications of Biodegradable Polymers in Electronics, Sensors, Actuators, and Healthcare

**DOI:** 10.3390/ma18194485

**Published:** 2025-09-26

**Authors:** Reshma Kailas Kumar, Chaoying Wan, Paresh Kumar Samantaray

**Affiliations:** 1 Department of Chemical and Materials Engineering, The University of Alabama in Huntsville, Huntsville, AL 35899, USA; 2 International Institute for Nanocomposites Manufacturing, WMG, University of Warwick, Coventry CV4 7AL, UK

**Keywords:** biodegradable polymers, transient electronics, resorbable biosensors, soft actuators, bioresorbable implants

## Abstract

Biopolymers have emerged as a transformative class of materials that reconcile high-performance functionality with environmental stewardship. Their inherent capacity for controlled degradation and biocompatibility has driven rapid advancements across electronics, sensing, actuation, and healthcare. In flexible electronics, these polymers serve as substrates, dielectrics, and conductive composites that enable transient devices, reducing electronic waste without compromising electrical performance. Within sensing and actuation, biodegradable polymer matrices facilitate the development of fully resorbable biosensors and soft actuators. These systems harness tailored degradation kinetics to achieve temporal control over signal transduction and mechanical response, unlocking applications in in vivo monitoring and on-demand drug delivery. In healthcare, biodegradable polymers underpin novel approaches in tissue engineering, wound healing, and bioresorbable implants. Their tunable chemical architectures and processing versatility allow for precise regulation of mechanical properties, degradation rates, and therapeutic payloads, fostering seamless integration with biological environments. The convergence of these emerging applications underscores the pivotal role of biodegradable polymers in advancing sustainable technology and personalized medicine. Continued interdisciplinary research into polymer design, processing strategies, and integration techniques will accelerate commercialization and broaden the impact of these lower eCO_2_ value materials across diverse sectors. This perspective article comments on the innovation in these sectors that go beyond the applications of biodegradable materials in packaging applications.

## 1. Introduction

The ubiquitous presence of plastic and electronic devices in modern society has led to a dual crisis: the escalating accumulation of plastic waste and the global surge in electronic waste (e-waste). Each year, global plastic production exceeds 390 million metric tons, with a significant subset entering landfills or the natural environment, persisting for centuries, and fragmenting into microplastics that infiltrate the food chain and threaten ecosystems and human health [1]. Concomitantly, e-waste is increasing at an alarming rate. In 2022, global e-waste reached 62 million tons, an 82% rise since 2010, with only about 22% formally recycled [2]. The convergence of these environmental and health concerns has catalyzed an urgent quest for sustainable material solutions, especially in the design and fabrication of next-generation electronic devices, sensors, actuators, and medical technologies.

Traditional plastics and electronic materials are primarily derived from fossil resources, are non-renewable, and are inherently resistant to degradation [3,4]. Beyond their persistence, conventional electronics typically incorporate toxic heavy metals and halogenated flame retardants. This intensifies the environmental impact when these products reach the end of life, often resulting in hazardous leachates that contaminate soil and water, or air pollutants released by incineration [4,5]. Inappropriate management and disposal raise public health risks and accelerate ecological degradation, challenging the very foundation of circular economy principles and sustainable development. Biodegradable polymers constitute a diverse family of macromolecules distinguished by their ability to undergo fragmentation, assimilation, and mineralization into non-toxic byproducts through chemical, physical, or biological processes. They can be classified according to their origin (natural vs. synthetic), chemical structure, or application-specific properties [6,7]. Natural Biopolymers are produced by living organisms or derived from renewable bioresources. These include polysaccharides (cellulose, chitosan, alginate, starch), proteins (collagen, gelatin, silk fibroin), and polyesters (polyhydroxyalkanoates, or PHAs) [8,9]. Natural biopolymers are inherently biocompatible, making them attractive for biomedical and environmental applications; however, they often exhibit high batch-to-batch variability and limited tunability of their mechanical and electrical properties. Synthetic Biodegradable Polymers, on the other hand, are synthesized via industrial polymerization processes; these materials offer more consistent physicochemical properties and tailorability. Common examples include polylactic acid (PLA), polyglycolic acid (PGA), polycaprolactone (PCL), poly(butylene succinate) (PBS), and poly(lactic-co-glycolic acid) (PLGA). Their degradation is mainly mediated by hydrolyzable ester, anhydride, or amide bonds, and their rates can be adjusted via copolymer composition, crystallinity, or molecular weight [6,10,11]. Regardless of their origin, these materials play a crucial role in emerging electronics and healthcare applications. Table 1 presents some representative applications of these materials. Traditionally, these polymers lack the conductive properties for integration in electronics, sensing, and actuation. Conductivity is achieved by dispersing conductive additives, such as carbon nanotubes (CNTs), graphene, polyaniline (PANI), polypyrrole (PPy), metallic nanoparticles, or ionic salts, into the biodegradable polymer matrix. This approach creates percolative pathways for efficient charge transport while leveraging the host polymer’s biodegradability [12,13].

Life cycle assessment (LCA) measures the environmental impact of products throughout their lifecycle [14]. However, most biopolymer LCAs focus mainly on global warming potential and fossil energy, making biopolymers seem more environmentally friendly due to their biogenic carbon [15]. This ignores key trade-offs like land use, eutrophication, water use, and toxicity. End-of-life (EoL) modeling is often simplified or omitted, despite its critical influence on decisions like composting, anaerobic digestion, recycling, or landfilling. According to the International Organization for Standardization (ISO), Life Cycle Assessment and its applications are defined in ISO-14040 and ISO-14044 [14]. The ISO’s LCA framework involves a four-step process: (1) defining the goal and scope, (2) analyzing inventory, (3) analyzing impact, and (4) interpreting the results. To provide accurate inventories for all processes related to a product’s production, use, and end-of-life, LCAs require extensive data. A comprehensive view requires combining impact categories with realistic EoL scenarios. The fate of materials at the end of their life (EoL) can substantially influence the results of conventional plastics and warrants equal consideration within biopolymer LCAs [16]. However, the reported results are not always directly comparable, as various studies tend to model diverse and sometimes non-realistic end-of-life scenarios, including landfilling, recycling, incineration, or composting [17]. This has caused recurring demand to conduct end-of-life in a deeper manner, despite the inventory data on biopolymer production and waste pathways becoming more refined and capable of being used to model them in greater detail. Thus, to mitigate a typical shortcoming, it would be preferable to explicitly model realistic EoL scenarios, such as landfill, industrial composting, and recycling. This will be a true indicator of the fact that the disposal options would have significant effects on the total life-cycle implications of such materials. Waste electrical and electronic equipment (WEEE, or e-waste) has become one of the fastest-growing global waste streams, reflecting society’s increasing demand for digital devices. The European Union and other jurisdictions have established comprehensive policy frameworks aimed at minimizing environmental impact, with a focus on repairability, reuse, recycling, eco-design, and principles of the circular economy. Nevertheless, in practical terms, the volume of e-waste generated continues to exceed the capacity of formal collection and recycling systems, highlighting the necessity for broader adoption and more effective implementation of these measures [18]. Over the past two decades, policy reforms have clarified responsibility and promoted eco-design and cleaner or greener treatment methods, thereby enhancing material recovery and alleviating ecological and resource-related concerns. However, recycling efforts remain inconsistent, with capacity still dependent on resource-intensive traditional techniques. Fourth-Industrial Revolution technologies, such as AI, Internet of Things (IoT), robotics, and advanced analytics, have the potential to enhance design, traceability, and recovery rates, provided that their deployment is equitable and regionally inclusive.

In this article, the term “biopolymers” refers to polymers derived from natural or renewable resource sources, such as PLA, starch, and cellulose. Biodegradable polymers like PCL and PHA are broken down through the action of microbial enzymes, which form environmentally benign end-products such as CO_2_, biomass, water in aerobic conditions, and methane in anaerobic conditions [14]. These properties are shaped by specific environmental conditions and testing standards because biodegradation depends on certain factors; for example, industrially compostable PLA cups meet EN 13432:2000, and soil-biodegradable mulch films comply with EN 17033:2018 [19]. On the other hand, biopolymeric materials are engineered, application-driven systems consisting of a single or more polymers combined with plasticizers, stabilizers, fillers, reinforcements, dopants, or coatings, such as a PCL/PLA blend or a cellulose-based composite. Their performance and end-of-life performance are, therefore, dependent on the comprehensive formulation and not only on the base polymer [6]. This perspective article intends to comment on the start of advances in biodegradable polymers for electronics, sensing, actuation, and healthcare applications. While doing so, we also want to highlight the significant prospects these polymers offer in these domains.

**Table 1:** Representative Biodegradable Polymers, Origins, and Key Properties.

Polymer	Source	Key Properties	Degradation Mechanism	Representative Applications	Limitations	Ref.
Cellulose	Plant	High strength, renewable	Enzymatic hydrolysis	Paper electronics, substrates, sensors	High reprocessing costs, moisture sensitive.	[20,21,22]
Chitosan	Animal	Biocompatible, antimicrobial	Enzymatic, hydrolytic	Wound healing, scaffolds, biosensors	Weak mechanical properties	[23,24]
PLA	Synthetic	Tunable, transparent	Hydrolysis	Packaging, 3D-printing, medical implants	Low melting point, Brittleness	[25,26,27,28,29]
PLGA	Synthetic	Sensitivity towards temperature, FDA-approved	Hydrolysis	Drug carriers, bioresorbable electronics	Production of acid upon degradation	[30,31,32,33,34]
PCL	Synthetic	Flexible, slow-degrading	Hydrolysis	Scaffolds, adhesives, sutures	Slow degradation	[35,36,37,38]
PHAs	Microbial	Elastomeric, hydrophobic, excellent mechanical properties	Enzymatic hydrolysis	Actuators, implants, drug delivery	Low flexibility	[39,40,41]

## 2. Advances of Biodegradable Polymers in Electronics Applications

Biopolymers must demonstrate a distinctive combination of mechanical, chemical, and electrical properties to be regarded as valuable in active electronic applications beyond passive utilizations. The intrinsic design and architecture, featuring a tunable elastic modulus and excellent tensile strength, provide significant flexibility and mechanical stability, which are essential for electronic components to function adequately as flexible electronics. The ability of chemical modification, surface engineering, specific absorption, ionic conductivity, and interfacial interactions of biopolymers, which necessitate the presence of functional groups such as hydroxyl, carboxyl, and amino, is vital for sensing and conducting functionalities. In addition, many biopolymers have inherent dielectric strength, conductivity, hydrophobicity, and biocompatibility, making them all well-suited towards implanted and wearable biomedical applications [42]. However, biopolymers suffer from limited thermal stability, and, hence, it is challenging to utilize them in high-temperature environments. Fundamental thermal properties such as glass transition temperature (T_g_), melting temperature (T_m_), and degradation temperature depend on their chemical structure, crystallinity, and functional groups associated with the polymeric backbone. Despite these challenges, new methods of molecular engineering, including cross-linking, doping, and blending, have provided researchers with the ability to tailor these materials to multifunctional, high-performance bioelectronic applications. Cellulose nanocrystals (CNCs) derived primarily through sulfuric acid hydrolysis exhibit high crystallinity, mechanical strength, and low thermal stability due to the presence of surface sulfate groups. However, the incorporation of reinforcement agents has enhanced the time of onset of degradation and tolerance to heat [43].

Considering mechanical properties, most biopolymers derived from starch exhibit high hydrophilicity and water-absorption capabilities, which result in poor mechanical strength relative to synthetic polymers. Nevertheless, the addition of urea as a plasticizer in corn starch has been observed to exhibit better mechanical properties, whereby the higher the urea concentration, the stronger the film, implying both higher strength and elasticity in the plasticized properties [44]. The electrical properties of chitosan are relatively low in conductivity because it lacks mobile hydrogen ions in the polymer structure. However, doping with electrochemically active species (e.g., acids or inorganic salts) may further provide extra charge sites, which facilitate proton migration. In addition, the electrostatic interaction between the acid groups and the basic amine groups of chitosan also facilitates proton transportation, therefore, significantly contributing to proton conductivity in the chitosan membrane [45]. In this discussion, we elaborate on how these structural and functional characteristics are utilized and reengineered to meet the increasing demands of flexible and sustainable electronic technologies [46,47,48].

Starch-based biopolymer gel electrolytes have gained significant attention due to their simple fabrication process and cost-effectiveness. Finkenstadt et al. [49] found that incorporating metal halides into extruded starch films greatly improved electrical conductivity, increasing it from 10^−11^ to 10^−6^ S/cm. Hou et al. [50] observed that starch hydrogels formed with inorganic salt solutions achieved conductivities of 0.2–0.3 S/cm. Wang et al. [51] and Zhang et al. [52] demonstrated that plasticizing starch with ionic liquids, such as 1-allyl-3-methylimidazolium chloride and 1-ethyl-3-methylimidazolium acetate, can increase conductivity to 10^−3^ S/cm and 0.0118 S/cm, respectively. However, this often reduces mechanical strength because water content weakens the interactions between starch chains. Notably, Zhang’s study revealed a Young’s modulus of 3–4 MPa, whereas Hou’s findings suggested a value of approximately 1 kPa.

**Figure 1:**
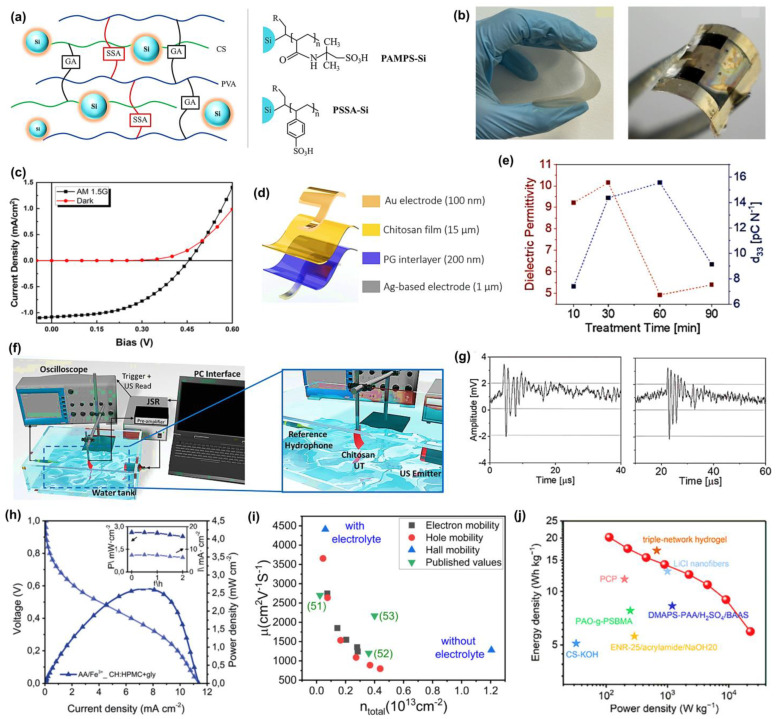
(**a**) Schematic illustration of CS/PVA IPN membrane system. Adapted with permission [55]. Copyright 2020 Elsevier. (**b**) Photograph of the CP membrane (**left**) and the device fabricated on the CP membrane (**right**). Adapted with permission [64]. Copyright 2025 American Chemical Society. (**c**) J-V characteristic curves for the device. Adapted with permission [64]. Copyright 2025 American Chemical Society. (**d**) Scheme of the device using a CS membrane. Adapted with permission [65]. Copyright 2023 Wiley-VCH GmbH. (**e**) Plot of dielectric permittivity and piezoelectric coefficient values over treatment time. Adapted with permission [65]. Copyright 2023 Wiley-VCH GmbH. (**f**) Diagram showing the setup for ultrasound measurements. Adapted with permission [65]. Copyright 2023 Wiley-VCH GmbH. (**g**) Ultrasound signal at 1 MHz. Adapted with permission [65]. Copyright 2023 Wiley-VCH GmbH. (**h**) Polarization curves for redox batteries. Adapted with permission [66]. Copyright 2019 WILEY-VCH Verlag GmbH & Co. KGaA, Weinheim. (**i**) Comparison of mobility and carrier concentration in CVD graphene on Si/SiO_2_. Adapted with permission [68]. Copyright 2025 American Chemical Society. (**j**) Comparison of Ragone plots for AQS and other reported SCs. Adapted with permission [70]. Copyright 2025 Wiley-VCH GmbH.

Chitosan (CS) is another renewable polysaccharide derived from the deacetylation of chitin. It has higher crystallinity and a greater chemical reactivity, which is contributed to by its molecular structure that is rich in three functional groups: hydroxyl (-OH), primary amine (-NH_2_), and ether (C-O-C) groups [53]. However, the pristine polymer displays low proton conductivity along with a lack of physicochemical stability, hence restricting its applicability in proton-exchange membranes (PEMs). Consequently, structural modification, i.e., doping, crosslinking, blending, and composite membranes, is required to reengineer it for optimal use in this field. Rosli et al. [54] prepared an enhanced proton conductor via a structurally modified CS derivative, N-methylene phosphonic chitosan (NMPC with polyvinyl alcohol (PVA), by adopting the solution-casting method. Following the same method, SiO_2_ was incorporated into NMPC/PVA membranes. The proton exchange behavior of the membranes is closely related to their proton conductivity, ion-exchange capacity (IEC), and water uptake. Increasing concentration of modified CS in the NMPC/PVA system enhances water uptake and increases proton conductivity, because of the large numbers of hydrophilic H-bonding -OH, -NH_2_, and -PO_3_H_2_ groups present that allow the formation of hydrated networks and subsequent proton transport. The addition of SiO_2_ also increases the water content to 55.7%, IEC to 0.56 meq/g, and proton conductivity to 5.08 × 10^−4^ S/cm at 100 °C because of the presence of more proton-transporting pathways provided by the interaction of -Si-OH and by increases in hydration. A similar study by Kamjornsupamitr et al. [55] also utilized CS/PVA and sulfonic acid decorated SiO_2_ nanoparticles to fabricate an interpenetrating network (IPN) for low-cost proton conduction. They prepared CS and PVA crosslinked with sulfosuccinic acid (SSA), combined with glutaraldehyde, as shown in Figure 1a, where each plays a specific role: SSA as a proton source, glutaraldehyde was also added to minimize the water uptake and increase the mechanical strength. Further improvements to increase proton conductivity and hydrolytic stability, poly(2-acrylamido-2-methyl-1-propanesulfonic acid) (PAMPS-Si) or poly(styrene sulfonic acid) (PSSA-Si) nanoparticles were also added to the polymer matrix. The proton conductivity of CS-PVA-1.5SSA-GA reached a maximum of 1.1 × 10^−3^ S/cm. The effects of loading different nanoparticles (both PAMPS-Si and PSSA-Si) on water vapor absorption, water uptake, and IEC were not apparent. However, conductivity was also enhanced in the presence of nanoparticles, reaching a maximum of 3.83 to 3.9 × 10^−3^ S/cm in the composite membranes. The presence of a polymeric shell did not have a significant effect on proton conductivity in this work since comparable percentages of polymer weight (4.0% and 5.1%) were applied to the SiO_2_ particles, and the water uptakes of these composite membranes at the same loading were significantly comparable. Di Franco et al. [56] used ionotropic gelation to fabricate a PEM that showed excellent proton conductivity at low temperature, which makes use of CS and silicotungstic acid for H_2_-O_2_ fuel cells. These membranes functioned successfully as proton conductors in a low-temperature (25 °C) fuel cell system fueled on hydrogen with a power density maximum of 268 mW/cm^2^ and a Pt loading of 0.5 mg/cm^2^. Another group has studied cellulose nanocrystals (CNCs) for biopolymer-based fuel cell applications [57]. The protic ionic liquid (PIL) combined with imidazole was introduced into CNCs to produce self-standing and strong membranes through the simple solution casting procedure. These composite membranes have a high proton conductivity range 10^−4^–10^−3^ S/cm at 160–120 °C, which is essential to proton exchange at high temperatures for membrane fuel cells (HT-PEMFCs). According to the conductivity, these membranes are comparable to membranes made from synthetic polymers. Additionally, the temperature range for optimal performance of CNC/Im/PIL exceeds the target temperature for future non-humidified fuel cell devices. The simultaneous presence of imidazole and imidazolium in the PIL, along with the lower activation energy measured, indicates that these materials are suitable for proton conduction. The principles behind the proton conduction mechanism are proposed to be hydrogen bonding and ionic mobility in the constrained nanostructure of CNCs and the PIL. Their investigation on a nanoscale level showed that PILs tend to alter the crystallites, which could be due to a direct interaction between the imidazolium cations and the biopolymer. Zhang et al. [58] suggested an alkali-resistant hydrogel electrolyte membrane prepared through the graft copolymerization of a carboxylated chitosan hydrogel. Compared to PAAS electrolyte, the prepared membranes have better properties, including high ionic conductivity 7.82 × 10^−2^ S/cm, and high electrolyte uptake of 524%. The alkaline supercapacitor has a commendable energy density of 4.39 Wh/kg and a power density of 224.99 W/kg in a quasi-solid state. They explained that these biopolymeric gel electrolytes can also be utilized in acidic and neutral electrolytes.

In the case of starch-based hydrogels, their mechanical properties are usually limited due to the rigid and highly branched nature of the amylopectin structure, which hinders intermolecular interactions. However, amylose-rich starches have more linear chains, resulting in stronger hydrogen bonding and entanglement, and, therefore, superior mechanical properties [59]. Recent developments of high-amylose starch with calcium chloride have worked, creating flexible and strain-responsive hydrogels that can be used as wearable sensors [60]. The addition of inorganic salts, such as CaCl_2_, MgCl_2_, and ZnCl_2_, also increases plasticity and provides mobility of ions, which significantly enhances electric conductivity, a factor crucial in flexible electronics [61]. Based on these reports, Ma et al. [62] developed high-amylose-rich starch using CaCl_2_ and glycerol, which exhibited improved mechanical resilience (elongation at break of ~208%) and potential applications in flexible bioelectronics. Moreover, this starch-based Zn-Cu battery-based system is also a self-powered wearable sensor that can detect minute motions of the human body, such as a pulse on a wrist and throat vibration under compressive stresses, with a high sensitivity of 1.5371 kPa^−1^.

Saha and coworkers [63] have examined porous polylactic acid (PLA)/polyethylene glycol (PEG) films, 25:75 wt% as separators for supercapacitors, with ionic conductivities determined at room temperature to be 1.1 times 10^−1^ S/cm and 0.6 times 10^−2^ S/cm in 1 M H_2_SO_4_ and 1 M Na_2_SO_4_ inert electrolytes, respectively. The porous PLA film was prepared through a phase inversion process, and the scanning electron microscope (SEM) cross-sectional images showed a porous interconnected structure to facilitate ionic diffusion. Radio frequency (RF) treatment, which uses air plasma, was used to enhance wettability by modifying the separator surface. A sustainable organic photovoltaic was prepared by das Neves et al. [64] by the casting and slot-die printing technique. In the casting approach, chitosan was combined with a polysulfone solution, after which they were cast onto a substrate to form flexible membranes, as shown in Figure 1b, left, and an optoelectronic device membrane, as shown in Figure 1b, right. For the slot-die method, the chitosan solution was printed onto a heated substrate at 70 °C, resulting in thin (~10 µm) and flexible films without the need for any additional polymer or polysulfone solution. The membrane has the characteristics of a rectifier diode, short-circuit current of 1.1 mA/cm^2^, and open-circuit voltage of 0.45 V during illumination in darkness (black squares) and under light (red circles), shown in Figure 1c. It can be successfully combined with conductive polymers (e.g., PEDOT:PSS), which permits making efficient charge transport interfaces without sacrificing the expense and brittle nature of transparent electrodes like ITO. Moreover, the optical transmittance (>70%) and mechanical stability of the chitosan-based membrane can be easily restored after repeated bending, making it an interesting green candidate material for the flexibility and printability of photovoltaic devices. Another interesting study using CS demonstrated a simple method to enhance the piezoelectric coefficient. The first piezoelectric transducers based on chitosan were demonstrated by de Marzo et al. [65] They produced a transparent chitosan film, giving a piezoelectric coefficient of 15 pC/N, which is more than twice the reference value of 6 pC/N. They develop the initial prototypes of a flexible ultrasonic transducer (UT) with a high sensitivity of 80 mV/kPa and a semi-transparent pMUT chitosan. A stretchable, flag-like UT was fabricated using chitosan films modified with a pair of asymmetric electrodes of different types, gold (Au) and silver (Ag), referred to as CT-Au/Ag, as shown in Figure 1d. The first step in the fabrication process was 3D printing of silver nanoparticle ink, printed using LDM printer on glass, subsequently spin-coated with 200 nm thick PEDOT:PSS mixed with 4% glycerol to make the material conductive and adhesive. Films were cast from a chitosan solution over the treated surface and air-dried. The result of the electrical characterization was that the capacitance of all samples was in the nanofarad range and decreased at higher frequencies (2 Hz to 1 MHz). They compare dielectric permittivity and piezoelectric coefficient versus time to calculate the figure of merit, as shown in Figure 1e. The highest figure of merit was attained at 5.6 GPa, suggesting that the electromechanical conversion efficiency was better. The maximum sensitivity like 80 mV/kPa was observed with the maximum output of the device like 32 mV and the linear working range being 0–0.5 kPa. This is one of the highest sensitivities reported for both biodegradable and conventional flex piezoelectric transducers. In particular, it was responsive to finger tapping (1.25 kPa), and human blowing (25 Pa), which are relevant pressure ranges. The immersion transducer (1 MHz frequency) was used to analyze the ultrasonic sensing as depicted in Figure 1f. All parts were immersed in a water tank to enable propagation of ultrasounds. Figure 1g shows the signal registered with CT-Au/Ag (left) and the ultrasound signal at 1 MHz registered with (right) pMUT, the measured peaks-to-peak output voltages after amplification, which were 5.3 mV in CT-Au/Ag and 5.4 mV in pMUT. The maximum range at which all devices could detect the ultrasound signal was 10 cm, with a Time-of-Flight (ToF) of ~70 µs. Alday et al. [66] explored environmentally benign biopolymer electrolyte membranes (BioPEMs) made of chitosan (CH), cellulose (hydroxypropyl methylcellulose, HPMC), and starch, which is comparable in performance to Nafion™ (DuPont, Wilmington, DE, USA) membranes. BioPEMs based on chitosan-cellulose (CH:HPMC) doped with ionic liquids (ILs) have successfully found application in primary redox batteries. Crossover experiments involving eco-friendly redox species: hydroquinone sulfonic acid (H_2_BQ), ascorbic acid (AA), p-benzoquinone (p-BQ), as well as Fe^3+^ were conducted to reveal that CH:HPMC+gly was of high selectivity to limit species migration, especially species with charge. They subsequently tested these BioPEMs in battery-based systems with two different redox chemistries (AA/Fe^3+^ and H_2_BQ/p-BQ), which showed a high open circuit voltage (0.75 and 0.90 V) and specific power densities of up to 2.5 mW/cm^2^ at greater than 90% stability over time, as shown in Figure 1h. Prajapati et al. [67] provide a detailed study of biopolymer blend electrolyte membranes (BPBEs) composed of chitosan, PVA, and PEG-200, doped with different concentrations of Mg(NO_3_)_2_, for use as an electrode in electric double layer capacitors, EDLCs. Electrical characterization of the membranes showed that the optimized composition (BPBE-4, 30 wt% Mg(NO_3_)_2_) had the highest bulk dc conductivity of 1.20 × 10^−4^ S/cm at 30 °C with the ionic transference number of 0.99, indicating that it was ion-dominated conduction. The sample also exhibited the lowest activation energy of 0.20 eV, indicating efficient ionic transport due to the increased amorphous content. AC conductivity obeyed the Jonscher power law and the power exponent 0.60 s, whereas the dielectric analysis showed a single relaxation peak corresponding to polymer chain dynamics. The membrane was determined to have an ion diffusivity of 1.33 × 10^−1^ cm^2^/s, charge carrier density (N) as 1.48 × 10^21^ cm^−3^, and the mobility (µ) as 5.08 × 10^−7^ cm^2^/V.s. Linear sweep voltammetry also determines an electrochemical stability window between −2.0 and 2 V, verifying that it is suitable to use with EDLC devices. The peak specific capacitance, energy density, and power density of the optimized BPBE membrane when assembled into an EDLC were found to be 6.05 F/g, 2.26 Wh/kg, and 61.42 W/kg, respectively, at 1.5 V and 0.5 µA/cm^2^, indicating its potential use as a sustainably high-performance energy storage device.

The use of solid biopolyelectrolytes with 2D materials has not been extensively examined. Alshehri et al. [68] solve the critical problem of electronic waste with biodegradable and renewable biopolymers capable of forming stable electric double layers (EDLs), permitting effective gating at low voltage (<1 V) and high mobility ~5000 cm^2^/V.s. Moreover, this work reports on gate-dependent EDL capacitance of monolayer graphene devices, effectively distinguishing the effect of quantum capacitance of graphene and EDL capacitance. They used chitosan, xanthan gum, and sodium alginate. Its rational formulation involved glycerol plasticization to increase the film’s flexibility and raise its ionic conductivity. Chitosan-based transistors demonstrated significant improvement of the hall mobility of 1200 cm^2^/V.s (without chitosan) to 4411 cm^2^/V.s (presence of chitosan membrane) and reduction in the carrier concentration of 6.1 × 10^11^ cm^−2^ with electrolyte gating, shown in Figure 1i. Such improved performance is due to the screening effects of the layer of chitosan. It confirms gate dielectric performance by field-effect and Hall measurements and makes these biopolymers viable options for low-power applications in eco-friendly, scalable electronics. Tang and coworkers [69] have developed an RCC composite membrane that functions as both a separator and an electrode for supercapacitors using a one-pot approach, comprising reduced graphene oxide (RGO), cellulose nanocrystals, and cellulose nanofibers. The hydrophilicity and the mechanical properties of RCC composite membranes were more favourable than those of traditional membranes. Furthermore, the specific capacitance of the RCC composite membrane was 171.3 F/cm^3^. As a result, a symmetric and flexible supercapacitor in solid-state (FASC) was fabricated using nanocellulose, and a porous cellulose membrane was used as a separator. This exhibited 164.3 F/cm^3^ of specific capacitance and an energy density of 3.7 mWh/cm^3^ that surpassed FASCs that have ever been reported. Recently, Li et al. [70] demonstrated a sustainable approach toward the fabrication of supercapacitors by utilizing sodium alginate (SA) via in situ carbonization of Ca^2+^ crosslinked alginate with urea. They obtained a remarkable gravimetric capacitance of 233.6 F/g and an impressive areal capacitance of 29.2 µF/cm^2^ at a current density of 0.25 A/g at 6 M KOH. The device exhibits a high energy density of 20.2 Wh/kg or 112.5 W/kg, which is maintained at 6 Wh/kg at 22.5 kW/kg, with 95.9% cycling stability over 10,000 cycles at 10 A/g, as shown in Figure 1j. This alginate-based system makes biomass-based materials for energy storage a reality.

However, biopolymer-based electronics have a major limitation to thermal stability, with heat and humidity potentially causing softening, hydrolysis, and interfacial degradation that can negatively affect electrical and mechanical performance. Zhu et al. [71] have introduced chitosan-hydroxyapatite aerogels, demonstrating an effective stabilization method. Covalent crosslinking and freeze drying yield anisotropic porosity to form a honeycomb network. This leads to a low radial thermal conductivity of 28.16 to 37.43 mW/m·K, slower degradation, higher char yields at 800 °C, and reduced heat release rates during combustion, while maintaining high porosity and a reinforcing matrix. These properties collectively serve as a thermal shield, providing a viable blueprint for bioelectronic substrates or encapsulants that can withstand changes in temperature. In light of this evidence, design principles aim to enhance the softening of the window through the use of crosslinking biocompatible and thermally stable fillers, which improve char formation, directional heat pathways, and ensure property retention at operating temperatures and relative humidity levels, in addition to conventional thermal properties. Similarly, Darius et al. [72] engineered a biosensor with encapsulation that can maintain the functionality of the device at high temperatures by overlaying a 2D perovskite with silk-fibroin. Under 40 °C, the biosensor maintained approximately 85% sensitivity after 1–2 days and 57.8% sensitivity after 7 days, in contrast with ~13% without silk. Notably, performance at the sensing level (LOD 10 pg/mL of cTnI) remained, and the film could be washed off after storage to measure it. This exhibits, with a benign biopolymer barrier, a low-energy, cold-chain-free route to thermally robust bioelectronics. In the case of biopolymer energy-storage devices, the thermal stability of the electrolyte plays a vital role in device safety, shelf life, and capacity sustainability during heating processes associated with operation and charging. In a recent study, Naveen et al. [73] investigated a solid polymer electrolyte based on gelatine and PVA doped with MgCl_2_. They discovered that the higher salt level of 8 wt% forms a more amorphous structure, which yields a 4.1 × 10^−3^ S/cm ionic conductivity at room temperature. TGA curves from 0 to 350 °C show that the main mass loss begins around 200–220 °C, indicating sufficient thermal stability of low-voltage EDLCs. Additionally, the optimal film has the lowest activation energy of 0.21 eV in the Arrhenius analysis. The gelatine/PVA/MgCl_2_ system is a potential candidate in green supercapacitors under the condition that the operating temperature does not reach the point of thermal degradation. As such, in general, with benign encapsulation, crosslinking or stereocomplexation, and thermally stable fillers, biopolymer electronics can attain the thermal profile required to ensure reliable operation in real-world temperature variations and end-of-life sustainability.

Thus, biopolymers have proven versatile in sustainable electronics due to their tunable physicochemical, electrical, and mechanical properties through various structural modifications. Applications in proton exchange membranes based on chitosan, as well as the ionic conductivity of cellulose, supercapacitors constructed from alginate, and PLA-based electronics demonstrate that these materials hold great promise for next-generation electronic, energy, and sensing systems. As molecular engineering and surface modification of biopolymers continue, biopolymers are expected to dominate the world of flexible, high-performance, and low-energy-consumption electronic systems. A comparative study of biodegradable polymer composites and their key characteristics is provided in Table 2.

## 3. Advances of Biodegradable Polymers in Sensing Applications

The characteristic features of biopolymers make them highly desirable in sensor development due to their biocompatible, biodegradable, functional group versatility, and sustainable nature. In wearable, flexible, or electrochemical sensing applications, it is crucial that the materials are mechanically stable, exhibit excellent hydrophilicity, have high adsorption capacity, and possess chemical sensitivity. Additionally, incorporating reactive functional groups, such as hydroxyl, amine, and carboxyl groups, is beneficial. These attributes help immobilize bioreceptors, promote interactions with their targets, and promote effective signal transduction [74]. Despite potential limitations such as low solubility and thermal stability, biopolymers like starch, chitosan, cellulose, and PVA are often combined with conductive additives like metal oxides, carbon nanotubes, or conducting polymers to address these issues. Compared to synthetic or carbon-based alternatives that depend on possibly toxic processes, biopolymer composites are a more environmentally friendly option for developing sensitive, selective, and multifunctional biosensors.

**Figure 2:**
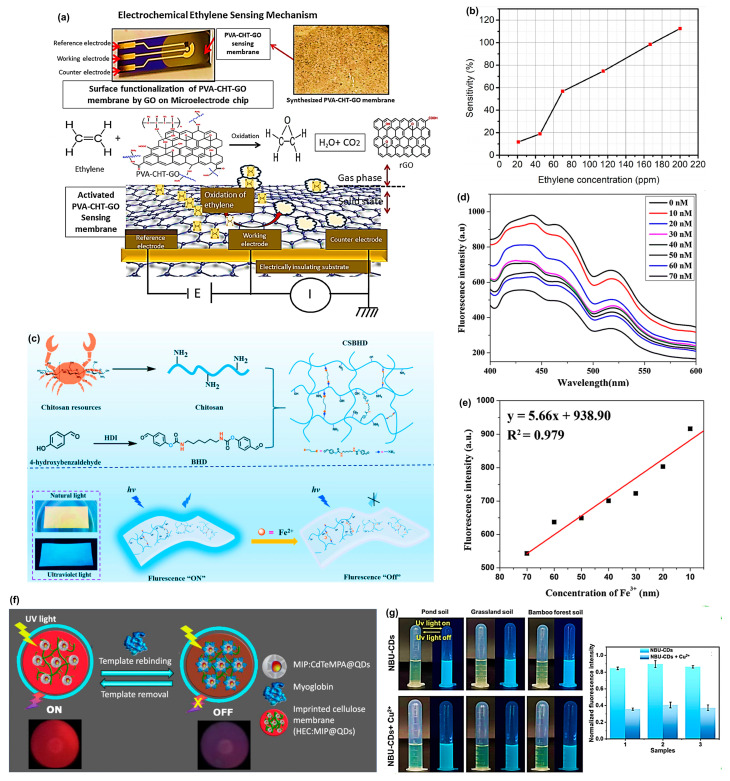
(**a**) Electrochemical ethylene sensing mechanism. Adapted with permission [75]. Copyright 2024 Elsevier. (**b**) Effect of ethylene concentration on detection sensitivity. Adapted with permission [75]. Copyright 2024 Elsevier. (**c**) Schematic representation and detection mechanism of the CSBHD hydrogel membrane probe. Adapted with permission [76]. Copyright 2024 Elsevier. (**d**) Fluorescence spectra of PCL/7.5FPN. Adapted with permission [77]. Copyright 2019 Springer. (**e**) Calibration curve for LOD calculation. Adapted with permission [77]. Copyright 2019 Springer. (**f**) Schematic representation of the assembly of the imprinted cellulose membranes. Adapted with permission [91]. Copyright 2021 American Chemical Society. (**g**) Fluorescence colour change and fluorescence intensity change observed in the soil extract solution mixed with NBU-CDs for Cu^2+^ detection. Adapted with permission [92]. Copyright 2023 Elsevier.

The biodegradable membrane, with chitosan (CHT), polyvinyl alcohol (PVA), and graphene oxide (GO), was first reported by Roy et al. [75] to address the urgent need for high-sensitivity ethylene detection, shown in Figure 2a. PVA is a strong film-forming polymer network with hydrophilic characteristics, and chitosan has high chain mobility and rich amine groups, allowing for extended interaction with GO and gaseous molecules. GO insertion into the PVA-CHT mixture, and subsequent aqueous GO-assisted surface activation resulted in a large enhancement of the oxygenated functional group and defect density and, therefore, allowed rapid charge transport and higher ethylene adsorption. Such an arrangement allowed redox interaction between ethylene and the GO-activated composite. The analysis of the cyclic voltammetry (CV) also found that prior to exposure to ethylene, the membrane had an anodic peak and a cathodic peak of potentials of 0.05–0.10 V and 0.02–0.13 V, respectively. After the introduction of 180 ppm ethylene, the anodic peak shifted towards 0.03 to 0.07 V, and peak currents were considerably reduced at all applied potentials, meaning that ethylene was quickly oxidized and the electrons were transferred through the GO layers. The decrease in ethylene concentration (from 30 ppm) exhibited gradual changes in peak amplitudes that persisted for more than 100 min. Conversely, at a higher concentration (180 ppm), the disappearance of peaks occurred within 30 min, with faster saturation levels resulting from a stronger interaction between ethylene and graphene oxide (GO). The membrane exhibited better sensitivity, which was calculated up to 200 ppm of ethylene at ~80% using the GO-activated PVA-CHT-GO membrane, as shown in Figure 2b.

A similarly important category of sensors includes portable and biodegradable membranes used for detecting heavy metals. Wei et al. [76] explored a fluorescent hydrogel membrane based on chitosan (CSBHD) for the detection and adsorption of ferrous ions (Fe^2+^). Chitosan was crosslinked with bis(benzaldehyde) (BHD) to form a Schiff base hydrogel that exhibits significant fluorescence, as shown in Figure 2c. The CSBHD membrane emits intense blue fluorescence over a wide range when excited by 405 nm and emits at 475 nm. It exhibited a linear dynamic response of 0–160 µM accompanied by a great limit of detection (LOD) of only 0.55 µM in a selective quenching reaction with Fe^2+^. The adsorption capacity of the hydrogel to Fe^2+^ was also found to be high, reaching 223.5 mg/g with a concentration of 500 mg/L. Similarly, Rijin et al. [77] designed a portable sensor utilizing PCL electrospun membranes by impregnating them with a fluorophore, 4,4′-fluoresceinoxy bisphthalonitrile (FPN). The membrane exhibits strong fluorescence quenching upon increasing concentrations of Fe^3+^ due to chelation by cyanide. Figure 2d indicates that the fluorescence signal with various metal ion solutions of Fe^3+^ at 414 nm gradually decreased with an increasing concentration of Fe^3+^ (10–70 nM), thereby confirming successful quenching. The linear calibration curve is shown in Figure 2e, allowing the calculation of the limit of detection (LOD), which was 2.94 nM, demonstrating that the membrane sensor is highly sensitive. Recently, Liu et al. [78] designed a chitosan-based fluorescence membrane for detecting and adsorbing Pb^2+^ in groundwater. The membrane can also be incorporated in a portable laser-induced fluorescence (LIF) spectrometer to perform on-site detection, with removal efficiency of over 99% and an adsorption capacity of Pb^2+^ of 247.6 mg/g, and it is primarily governed by chemisorption. It allows real-time results of Pb^2+^ detection in groundwater by using the smartphone-based RGB (Red, Green, Blue) colour analysis and a portable LIFs platform, with satisfactory results.

Wang et al. [79] prepared a flexible strain sensor utilizing PVA, lignin, and multi-walled carbon nanotubes (MWCNT). Lignin was added to improve the electrical conductivity, mechanical properties, and piezoresistive sensitivity of the PVA substrate. The resulting conductive paths created between the lignin-rich zones under the action of strain are highly adaptive to deformation and so are measured in changes in the resistance. It has the minimum and maximum gauge factor values of 137.3 and 2746.4, respectively, in the strain range of 0–160% and 160–240% with the capability of repeatability (>10,000 tensile cycles). A fast, responsive, and recyclable sensor based on N-doped carbon dots (NCDs) modified PVA membrane is constructed to detect arginine (essential amino acid) by Yang et al. [80]. This is because PVA interacts with CDs, causing a self-assembly process that forms clusters and results in an increase in fluorescence due to aggregation-induced enhanced emission (AIEE). In the same AIEE effect, Liu et al. [81] have designed a PVA-coated gold nanocluster membrane and applied it as a pH sensor. The nanoclusters were stabilized by the PVA, which enhanced their fluorescence to a large extent by AIEE. The addition of PVA to adenosine 5′-monophosphate-capped gold nanoclusters (AuNCs@AMP) increased the emission to 35.4% by enhancing emission because of more aggregation in a less polar environment, and the particle emission peak shifted to 380 nm. PVA and AMP exhibited strong hydrogen bonding between the hydroxyl and amino groups, which hindered molecular movements, resulting in a decrease in non-radiative decay and an increase in luminescence.

As a recent advancement in chitosan-based optical fiber sensors, they can selectively and significantly detect the presence of polyphenolic compounds, such as caffeic acid and chlorogenic acid. In the case of caffeic acid, researchers used a molecularly imprinted chitosan (MIP) membrane as the sensing probe of a bent optical fiber sensor [82]. The MIP membrane crosslinked with glutaraldehyde and caffeic acid molecules as the template created selective detection sites to enable selective solid phase microextraction. This setup demonstrated an exceptional detection limit of 5.2 ng/mL (tens of times more sensitive) as compared to the traditional HPLC-UV/Vis methods. The same group employed for chlorogenic acid utilized a bent optical fiber probe with a chitosan coating to leverage the inherent strength of hydrogen bonds and electrostatic attractions provided by the polymer [83]. An increased sensitivity of 2400-fold was achieved when this sensor reached a detection limit of 0.018 µg CGA/mL.

Kyung Hyun and coworkers [84] introduce a highly sensitive biodegradable pressure sensor using a nanofibrous dielectric membrane based on a composite of polylactic-co-glycolic acid (PLGA) and polycaprolactone (PCL). The use of PLGA gives it superior dielectric characteristics (dielectric constant ~3.3–4.4), whereas PCL makes it more mechanically flexible and processable. The sensor reported a very high sensitivity of 0.863 ± 0.025 kPa^−1^ at the low-pressure range (0 to 1.86 kPa) and 0.062 ± 0.005 kPa^−1^ at the higher-pressure range (1.86 to 4.6 kPa) with a low detection limit of 1.24 Pa. The degradation study indicated that it lost approximately 60% of its weight within 2 weeks and exhibited a 19.5% reduction in low-pressure sensitivity after one week of incubation, with minimal change in high-pressure sensitivity. In a recent study by Zhu et al. [85] investigated a flexible pressure sensor realized using a grid-patterned membrane made of PCL, thermoplastic polyurethane (TPU), and MWCNTs. Grid design significantly enhances the sensitivity of 2.24 kPa^−1^, with a response time of 9.17 ms and the response range of 105 dB compared to a planar sensor. The device accurately records all finger motions, and repeated tests during 1200 s of cyclic testing have shown excellent stability and durability; therefore, it portrays a significant potential for wearable electronics and gesture recognition. Liu and co-workers [86] present electrospun PLA membranes modified with CNT and layered double hydroxide (LDH), which can simultaneously serve as super oil-water separators and for real-time press sensing. This membrane forms a sea urchin-like structure that enhances dispersion and electrical conductivity. The material reduces the fiber diameter and pore size, increases hydrophobicity, and delivers high oil absorption (up to 35 g/g) and quick response to press-sensing. Li et al. [87] introduced reversible non-covalent crosslinks in the carboxymethyl starch, PVA, and silver nanoparticles (AgNP) to achieve a complete recyclable bioelectronics. They designed a sensor that can monitor small movements (drinking, speaking) as well as large movements (moving, jumping, elbow movements) with high sensitivity and cycling stability. The sensor shows excellent sensitivity (gauge factor as high as 4.02), high conductivity (0.1753 S/m), low detection limit (0.5%), and long-term antibacterial activities (more than 7 days).

Suvarnaphaet et al. [88] developed an electrode patch to monitor the electrocardiogram (ECG) based on a composite membrane consisting of graphene and polyhydroxyalkanoate (PHA) biopolymer, which aims to overcome the problems of skin irritation and the gel-based application of conventional Ag/AgCl electrodes. The rationale for this potential innovation lies in the combination of graphene’s electrical conductivity and biocompatibility with the flexibility and biodegradability of PHA. The last electrode patch had a resistance of 20 kΩ and in-circuit performance could sense normal (60 BPM, 1 mV amplitude) and abnormal ECG signals (AFIB, VFIB, VTACH) with an equally similar output to that of commercial electrodes. Particularly, electrodes based on graphene/PHA registered no signal drop during 100 cycles, and they successfully separated ECG signals with minimal noise, which affirmed the usefulness of their conductivity and mechanical strength. Notably, the patch also exhibited good signal clarity during limited bending, which points towards the possible implementation of the same in wearable and portable, environmentally friendly biomedical sensors.

A recent study by Vrážel et al. [89] demonstrates the application of PHA-based membranes as a potent tool for detecting aromatic hydrocarbons (BTX: benzene, toluene, and xylene) in aqueous solutions through mid-infrared (MIR) spectroscopy. Its detection principle is based on the diffusion and adsorption of BTX molecules onto ZnSe prisms coated with PHA, followed by their subsequent determination through ATR-FTIR spectroscopy. PHA membranes were prepared using the phase inversion process and then subsequently deposited either by knife or spin coating onto infrared-transparent substrates such as ZnSe prisms and chalcogenide glass-coated wafers. The detection was tested at 50 ppm in BTX solutions. PHA-based membrane demonstrates that only the low crystallinity samples, PHO (19% crystallinity) and amorphous P3HB4HB, exhibited the characteristic BTX absorptions in the fingerprint region (650–900 cm^−1^). More crystalline PHAs, including P3HB3HV (43% crystallinity), did not provide measurable peaks due to the limited diffusion pathways. Regeneration tests proved that this can be reused after simple washing with water. In this sense, the crystallinity of PHA remains a challenging criterion. Ren et al. [90] developed a fluorescent cellulose-based membrane sensor, aimed at highly selective detection of harmful toluene gas. The membrane was prepared by uniformly loading carbon quantum dots (CQDs) prepared via a green hydrothermal process using glycerol and betaine into a carboxymethyl cellulose matrix. The resulting membrane exhibited high fluorescence, with a quantum yield of 18.34% and a lifetime of 5.3 ns. There was a distinct linear fluorescence response due to an increase in toluene concentration (200 to 1400 ppm), and a low detection limit of 0.452 ppm was attained. Therefore, the sensor offers a highly inexpensive, visual, and environmentally friendly real-time platform for toluene detection in industrial and environmental settings.

Another interesting sensor was developed by Piloto et al. [91] a fluorescent biopolymeric membrane that was made up of molecularly imprinted polymer-coated cadmium telluride quantum dots (MIP@QDs) and hydroxyethyl cellulose (HEC) gel-based sensitizer that enables a pathway to specific and sensitive assay of cardiac biomarker myoglobin (Myo), shown in Figure 2f. Fluorescent quenching in tyrosinated membrane by the Myo showed a linear detection range of 7.39–291.3 pg/mL of 1000-fold diluted human serum and a limit of detection (LOD) of 3.08 pg/mL as compared to the clinical cutoff of 23 ng/mL of myocardial infarction. The specificity of a MIP-based recognition system was reaffirmed by an imprinting factor of 1.65, which was more than the controls of non-imprinting.

Recently, Cui et al. [92] suggested a biomass nanofiber membrane (NBU-CDs), which consists of xylan-derived carbon dots (U-CDs) in a blend of PLA and PCL, prepared through in situ electrospinning. The sensitivity of this novel membrane toward Cu^2+^ is truly impressive, with a detection limit of 0.83 µM. The binding between Cu^2+^ ions and the plasma surface -NH_2_ functional groups led to photo-induced electron transfer (PET), resulting in fluorescence quenching. The addition of NBU-CDs to soil extracts showed bright blue fluorescence, Figure 2g, and the addition of Cu^2+^ led to a dark colour (quenching), proving the qualitative detection. The inset bar graph in the figure indicates a significant decrease in fluorescence intensity after exposure to Cu^2+^. NBU-CDs also exhibited a high quantum yield (23.3%), rapid visual responses, and over 97–104% recovery in models of lake water, soil, and zebrafish. Mohammed-Sadhakathullah et al. [93] demonstrated the synthesis of nanostructured membranes composed of biodegradable PLA/PEG, in which a lipophilic molecule, cholesterol, is incorporated covalently. The PEG-cholesterol units exhibited a strong attraction to both hydrophilic binding materials, ascorbic acid (AA) and Trolox, with detection limits of 8.12 µM for AA and 3.53 µM for Trolox, respectively, in an aqueous salt solution. The bio-inspired membrane offers the opportunity to combine antioxidant capacity and facilitate the fabrication of anti-stress biosensors and electrodes for detecting vitamin C or vitamin E.

Joshi et al. [94] suggested that shellac could be used to derive reduced graphene oxide (rGO) films by heat treating to form the thermally decomposed rGO (TrGO) that had high carbon content, good crystallinity, and low sheet resistance. Label-free electrochemical immunosensors that detect H1N1 influenza were generated by dispersing TrGO flakes into indium tin oxide electrodes/glass with the help of drop-casting techniques to identify the specified influenza using electrochemical impedance spectroscopy. The sensors showed very high stability and reproducibility, presumably because the phenolic-OH groups provide a strong adhesion and reach detection limits of 26 and 33 PFU in phosphate-buffered saline and diluted saliva, respectively. This method highlights the prospect that TrGO could be a valuable, low-cost, dependable nanomaterial that could be used in biosensors in the field of clinical diagnostics.

**Figure 3:**
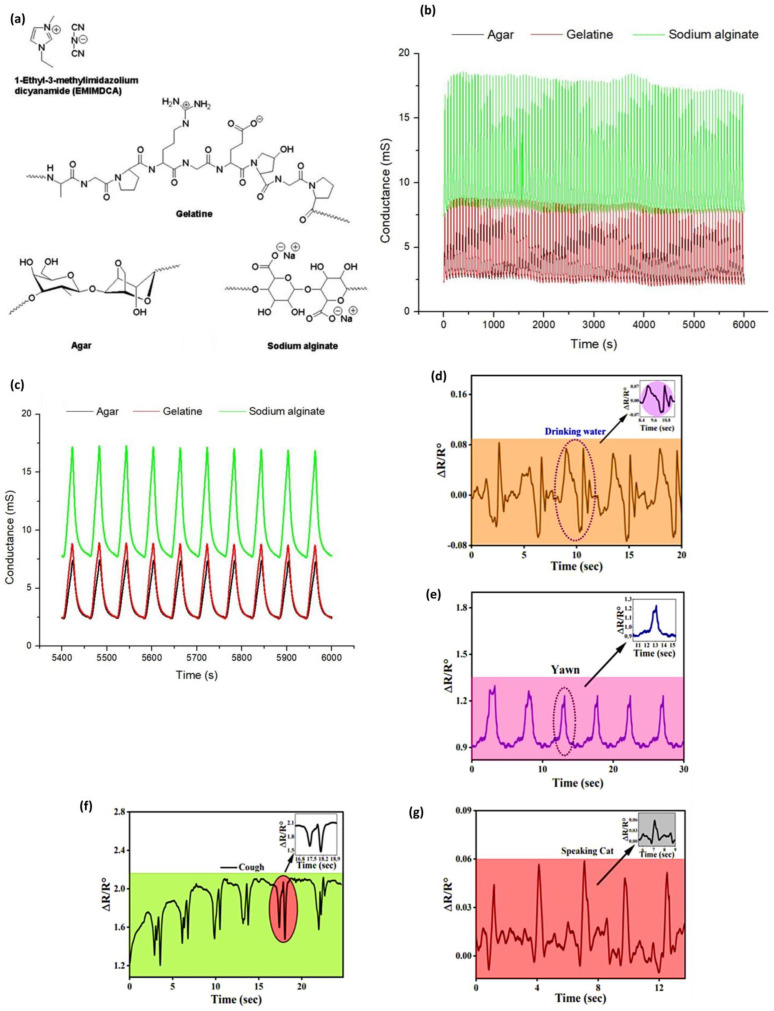
(**a**) Structural representation of ionic liquids and biopolymers. Adapted with permission [95]. Copyright 2020 Elsevier. (**b**) Sensors’ response to ethanol vapors. Adapted with permission [95]. Copyright 2020 Elsevier. (**c**) Last 10 cycles. Adapted with permission [95]. Copyright 2020 Elsevier. (**d**–**g**) The response of the hydrogel to minute physiological movements of the human body fixes on the vocal card: (**d**) water intake response, (**e**) yawn response, (**f**) cough response, (**g**) the hydrogel’s response to speaking cat. Adapted with permission [96]. Copyright 2024 Elsevier.

Jonas and coworkers [95] proved that biopolymers provided a structural network to entrap the ionic liquid to form stable ionogels and used them as the active sensing layer. The gelling agents, gelatin, agar, and sodium alginate, have formed H-bond and electrostatic interactions with the ionic liquid (EMIMDCA) and created a three-dimensional structure that ensured there was ionic mobility, but mechanical integrity was achieved to coat the interdigitated electrodes, the structural illustration of all biopolymers, including the EMIMDCA shown in Figure 3a. The vapors infiltrated the ionogel on exposure to volatile organic compounds (VOCs) and changed the ionic conductivity by influencing their polarity and dielectric environment. The change in conductance was recognized as a variation, and the relative response (Ra) was, therefore, computed according to the difference between the initial and the final conductance readings. The sensors showed specific responses to each solvent, and average Ra values would be used to distinguish them effectively. For example, the array achieved a classification accuracy of ≥96% for all VOCs, and in some cases, 100% using machine learning algorithms. Reproducibility was validated with relative standard deviations between 0.03 and 0.56 and long-term testing of over 100 cycles at 40 °C, as shown in Figure 3b, which showed stable responses in signal to show the ionic transport under the intact characteristic of the biopolymer-ionic liquid bonding. Figure 3c represents the final 10 cycles, indicating no signal drift, so that the biopolymer-ionic liquid network retained its conductivity and structure, thus ensuring that the sensor would be reliable in the long term.

Ara et al. [96] fabricate a wearable strain sensor based on gellan gum, polyacrylamide-co-butyl acrylate. It demonstrates remarkable strength in uninterrupted stretching and relaxing cycles without compromising its structural stability. Moreover, a broad sensing range and superior sensitivity to various strains, and a gauge factor (GF) of 31.51 were obtained in dual crosslinked hydrogel strain sensors. The conductivity was found to be 0.32 S/m, as NaCl is present in it. As such, the hydrogels act as wearable sensors at the vocal cord that can detect drinking, yawning, coughing, and the repeated pronunciation of the word cat. In Figure 3d–g, it becomes clear that the hydrogel was very helpful in monitoring both drinking water and the occurrence of yawning. The results also show that hydrogel consistently triggers the same reactions, followed by repeated drinking and yawning, and repeated pronunciation of the word cat. Interestingly Cui et al. [97] fabricated a multifunctional human movement monitoring sensor based on a starch/PVA system incorporated with CaCl_2_. Despite their poor freezing resistance and low electrical conductivity, starch exhibited remarkable stretchability of 478.1% and superior mechanical strength of 2.1 MPa. The sensor demonstrates high sensitivity (gauge factor = 0.74) and provides consistent performance over a broad strain range, making it ideal for real-time monitoring of physical activities and body temperature signals.

Biopolymers need to be designed to exhibit multiple characteristics, including mechanical resilience, high stretchability, good tensile strength, electrical conductivity, thermal stability, and environmental stability, as well as biocompatibility, to ensure the reliable performance of flexible and wearable sensing systems. Furthermore, properties such as fast response rates, sensing for volatile materials, anti-freezing efficiency, recyclability, and long operational time are crucial for real-time physiological monitoring and long-term use. These characteristics make the biopolymer-based sensors capable of withstanding repetitive mechanical deformations and retaining signal integrity. Recent research, which includes the use of plasticizers (e.g., CaCl_2_, glycerol), conductive fillers (e.g., graphene, CNTs), and crosslinking agents, has been effective in improving these characteristics. Thanks to these efforts, several biopolymer systems now show promise in competing with traditional materials in terms of gauge factors, conductivity, and durability, ultimately gaining credibility as biodegradable alternatives in wearable electronics. The comparative summary is provided below in Table 3.

## 4. Advances of Biodegradable Polymers in Actuation Applications

Compared to conventional dielectric elastomer actuators, which require high-voltage drivers and non-degradable materials, biopolymer actuators are more suitable for many applications due to their low-voltage operation, soft stimuli, and end-of-life compatibility [98]. Ionic and hygroscopic biopolymers can achieve large curvatures and effective blocking forces under typical environmental conditions, such as humidity or light. These devices are more energy-efficient because they eliminate the need for high-voltage electronics, which are common in systems like wearables and biointerfaces [99]. They are soft, tissue-like, hydrophilic, and have tunable crosslinking networks, leading to soft, tissue-like mechanochemical behavior, stability in wet environments, and natural biocompatibility—making them safe to interface with skin and biological fluids without encapsulation. It is important to note that biopolymers can be recycled or composted when they are properly designed, as they are also renewable. It enables actuators to provide the correct force and displacement to fit into particular applications with minimal material hazard, promote safer failure modes, and facilitates the benefits of the circular economy that traditional elastomeric actuators, despite their large maximum strain rate, cannot inherently offer. The important characteristic that actuator materials or composites must possess is unique reversible swelling/deswelling, deformation with respect to different stimuli (electric, magnetic, thermal, and chemical), excellent water retention, and shape adaptability, as well as durability to emerge as a potential candidate. Biopolymers, in particular, including chitosan, gelatin, alginate, and xanthan gum, which nanoparticles or crosslinkers may modify, fulfill these requirements due to the ability to deliver soft, safe, and programmable actuation [100]. They are responsive to electric fields, temperature, magnetic fields, or moisture, making them suitable for wearable health monitors, soft robotic grippers, artificial muscles, and drug delivery. The inclusion of biopolymeric microneedle patches and biomimetic adhesives can significantly enhance skin interaction, highlighting their potential application in flexible, responsive, and biodegradable actuating systems.

Xinhai He and coworkers [101] synthesized a biopolymer membrane consisting of chitosan (CS), polyvinyl alcohol (PVA), and azobenzene (Azo) using an evaporation-induced self-assembly technique, which was utilized as a controlled photo and humidity-responsive membrane. The membrane allows the extensive hydrogen bonding of CS, PVA and Azo to develop an effectively oriented surface structure with the use of a grooved silicon mold, in order to exhibit controllability of directional actuation upon light and humidity stimulus. The film prepared with 10 wt% Azo showed the maximum photoresponse under UV light (1 mW, 365 nm), as evidenced by a bending angle of 85° in 50 s. It bent to 144° under natural sunlight in 55 s and was moderately actuated under LED (5000 K). Under humidity (e.g., fingertip contact), the film could bend to 155 with a bending time of 30 s (2.5 wt% Azo), the 10 Azo film could bend to 140 and reverse to −180 under sunlight. These performances are owed to reversible cis-trans isomerization of Azo and the difference in swelling of the hydrophilic CS/PVA matrix. Moreover, the actuator could lift a staple (20 mg), 5 times as heavy as itself (4 mg), to a height of 12 cm in 75 s using sunlight, which is indicative of the conversion of light to mechanical energy. Hydrogen bonds on the exposed surface are disrupted by moisture, leading to the formation of new bonds with water. Consequently, the surface swells, a through-thickness gradient develops, and the film bends. Drying restores the original network structure, returning the film to a flat shape. Azo imparts photosensitivity, under UV or sunlight, it undergoes a conformational change from trans to cis on the Azo-rich surface, resulting in a local volume change that prompts rapid reverse bending. Increased Azo loadings result in microphase separation and enhanced stiffness, which diminishes the responses to humidity and light. Films devoid of Azo recover gradually following humidity exposure but do not revert to their bent form, thereby supporting the notion that recovery speed is improved by light-responsive behavior and facilitating reversible actuation. By blending with CS, the tensile strength and modulus of PVA were enhanced in the mechanical analysis due to the high intermolecular hydrogen bonding. However, when more than 10 wt% of Azo was added, phase separation occurred, resulting in a decrease in mechanical properties.

**Figure 4:**
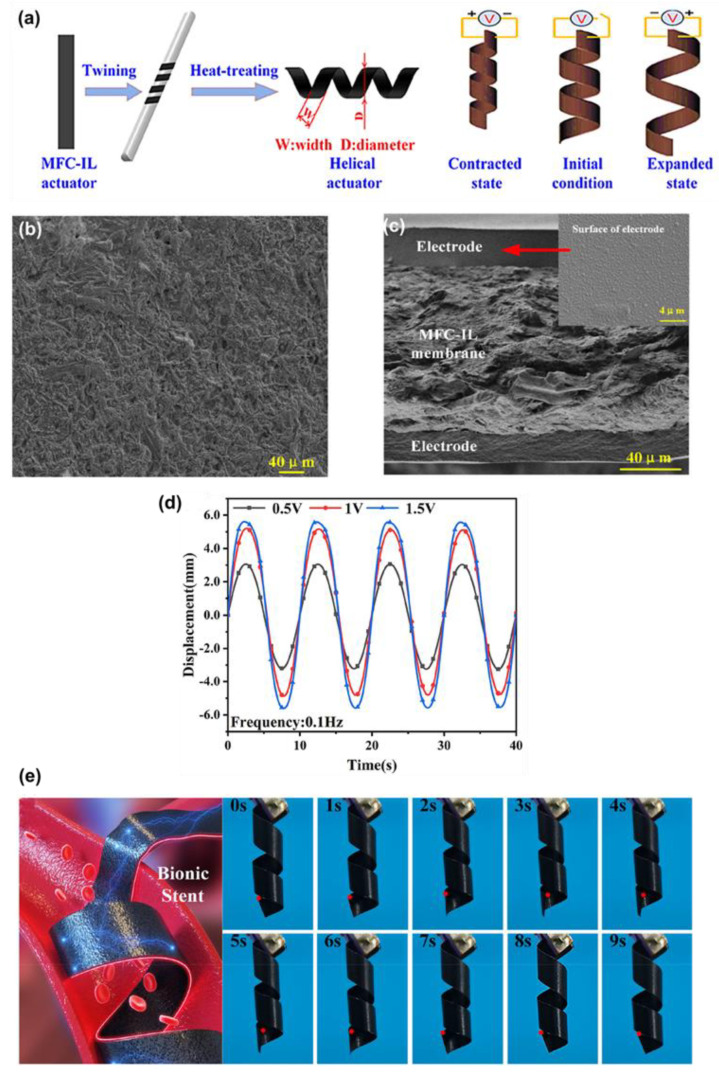
(**a**) Schematic representation of fabrication of the (MFC-IL) helical actuator. Adapted with permission [103]. Copyright 2022 Society of Plastics Engineers. (**b**) SEM images of the MFC-IL. Adapted with permission [103]. Copyright 2022 Society of Plastics Engineers. (**c**) Cross-sectional SEM image of the actuator. Adapted with permission [103]. Copyright 2022 Society of Plastics Engineers. (**d**) Actuator displacements are observed at different driving voltages. Adapted with permission [103]. Copyright 2022 Society of Plastics Engineers. (**e**) Optical images of the helical ionic actuator. Adapted with permission [103]. Copyright 2022 Society of Plastics Engineers.

Wang et al. [102] report a high-performance ionic electroactive polymer actuator (IEPA) synergistically built with carboxylated bacterial cellulose (CBC), ionic liquid (IL), and MWCNT and PEDOT:PSS electrodes. The actuator utilizes the excellent ionic interactions and crosslinking between CBC nanofibers, IL, and MWCNT, remarkably overcoming the drawbacks of traditional actuators. The actuator showed mechanical properties of Young’s modulus 349.1 MPa, and superior electrochemical properties with specific capacitance of 76.97 mF/cm^2^. They also showed that the CBC-IL-MWCNT actuator in biomimetic robotic fingers can be used to interact with touchscreen electronic devices. With the application of low voltage, the ions of the ionic liquid move and concentrate on the electrode interfaces, thereby creating electric double layers, and creating osmotic/pressure and electrostatic gradients across the thickness; at the same time, the electrochemical doping/de-doping of the PEDOT:PSS layers takes place. The redistribution of the asymmetric ions and the interfacial volume change generate a bending moment. The actuator had been tested under a low voltage of 1 V DC to conduct two specific human-like applications, namely, turning on and shutting down one timer application with exact timing at 10 s, and swiping across the display of a smartphone to change pictures. Similarly, Shen and coworkers [103] explored microfibrillated cellulose (MFC) for ionic soft actuator as shown in Figure 4a. The ionic interactions, especially those between hydroxyl moieties of the MFC and IL, strongly contributed to the ion transport through the membrane, as evidenced by the SEM images shown in Figure 4b. Figure 4c indicates the cross-sectional SEM image of uniform electrode adhesion. This interaction was verified using FTIR, where new characteristic peaks at 795 and 1259 cm^−1^ were detected, and XRD showed a reduction to 48.44%, signifying high amorphous regions which facilitate easy migration of ions. The MFC-based membrane actuator system demonstrated a high peak-to-peak displacement of 11.21 mm at an excitation frequency of 0.1 Hz, as shown in Figure 4d. It also exhibited low actuation voltage and superior durability (98% maintained after 2 h), along with controllability, due to the strong ionic interactions between MFC fibers and IL. The helical MFC-IL ionic actuator was further designed as a heat treatment, and it was possible that this actuator could be used to create a biomedical active stent that is in a position to control the radius of the actuators, which depends on changing the applied voltage and frequency (1.5 V sinusoidal excitation at 0.1 Hz), which is shown in Figure 4e. Thummarungsan et al. [104] designed a magneto-responsive smart actuator using poly(butylene succinate-co-butylene adipate) (PBSA) and integrating magnetite (Fe_3_O_4_) nanoparticles as an alternative to sustainable and non-toxic materials. This combination significantly enhanced both the magnetic sensitivity and mechanical strength of the actuator. When exposed to a non-uniform magnetic field, the Fe_3_O_4_ particles acquire dipole moments and experience magnetic forces or torque, causing them to align themselves with the field. The particles are incorporated within a compliant PBSA film, and via this field-induced particle interaction, a through-thickness body force forms, which bends the cantilever towards the magnet. The PBSA is soft, thus high curvature can be achieved; interfacial interactions facilitate load transfer, and small Fe_3_O_4_ loadings are tolerant to flexibility. The optimized amount with 3.0% *v*/*v* Fe_3_O_4_ shows a bending displacement of 15.6 mm in just 10 s. The actuator produced a magnetophoretic force (0.14 mN) in a relatively low magnetic field (1300 Gauss), with magnetization (1.719 emu/g), making it promising in low-field, fast-response soft robotics and biomedical applications.

Jaehwan Kim [105] explored an electrospun silk membrane coated with a sulfonated chitosan membrane. The investigation revealed that the material’s interdigitated structures had stable mechanical and electrochemical properties, making it suitable for fabricating high-performance ionic biopolymer actuators. Specifically, the introduction of sulfonated chitosan-ionic liquid decreases the mechanical stiffness of the electrospun silk. This enhances the ionic conductivity, causing the ion pathways within the interdigitated silk-chitosan domain. This actuator with the graphene-PEDOT:PSS electrodes exhibits a 165% bending enhancement. The proposed biopolymer actuator, with notable improvements, can be a candidate of interest for the formation of skin-attachable devices. The design of the carboxymethyl chitosan (CMCS) and the tannic acid (TA), by Fang et al. [106] involved the fabrication of an electrical response actuator of driver membranes using the vacuum freeze-drying method. Electrode membranes with sodium alginate (SA) and MWCNTs are produced via vacuum drying. Their findings revealed that the high output, specific capacitance, and strain in the absence of tannic acid solution are 1.95 mN, 68.36 mF/g, and 102.22%, respectively. When the amount of TA added to the samples is 14 mg, the maximum output force, specific capacitance, and strain of the samples increased to 6.12 mN, 99.82 mF/g, and 151.23%. The mechanism is examined through ion migration. The distribution of the driver membrane pores becomes increasingly uniform, and eventually, a large pore structure with filament crosslinking will form. This leads to improved driver and electrochemical performance.

Interestingly, Lv et al. [107] observe the effects of carboxymethyl cellulose (CMC) mass on the sodium alginate-carboxymethyl cellulose ion actuator (SCIA). They recorded its displacement on deflection and output force. Experiments indicated that the general performance of the SCIA sample was 0.25 g, which resulted in good output force, excellent channel of ions, and a fast response speed. The maximum electrochemical performance with force is 2.675 mN, dynamic speed is 0.176 mm/s, displacement is 17.137 mm, and the specific capacitance is 96.95 mF/g. Nevertheless, the performance of SCIA samples decreased as the CMC content increased and with excessive cross-linking. When exposed to an electric field, the SA/CMC network becomes a polyanionic gel: the remaining hydroxyl groups in SA are dissociated, and ions move to the anode, resulting in asymmetric anode ion accumulation and osmotic swelling, and cathode contraction. The resulting bending moment towards the cathode (cathode deflection behavior). Adding CMC creates a fiber network that dilates ion-transport channels and elevates specific capacitance, increasing the output force, deflection, and response speed to an optimal loading of CMC. It is vital to mention, though, that excesses of crosslinking may paralyze channels and considerably reduce performance. Instead, Glycerol plasticizes the network, assists in water retention, and is responsible for actuation stabilization.

Sodium alginate-PVA membrane obtained via freeze-drying was assembled on an electrode membrane to form a three-layer structure electric actuator (SPEA), prepared by Jia et al. [108] PVA is rich with hydroxyl groups, and sodium alginate has carboxyl and hydroxyl groups. PVA, when dispersed in water, has high hydrophilicity and can interpenetrate sodium alginate and construct a network structure, consequently destroying the original hydrogen bonds of sodium alginate. This enhances mobility between chains of the polymers. With PVA doping, the intermolecular forces are weakened, and sodium alginate can be easily deformed, which positively affects the output force. Nonetheless, excessive PVA may lead to a high rate of output force, which destroys the membrane and shortens SPEA’s lifespan. The sodium alginate network, when subjected to an electric field in NaCl, moves ions and charges the electric double-layer at the interfaces of the gel and electrodes. The resulting asymmetric ion build-up through thickness causes a bending force and a production force. PVA is a hydrogen-bonded network that interpenetrates sodium alginate, which is freeze-dried to form a porous 3D microstructure, which enhances water retention and ion movement, increases specific capacitance, and stabilizes actuation. The optimum amount of PVA for better performance was 1.0 wt%. The stable output force achieved at 5 V was 3.459 mN, 1.64 times stronger than that of the SPEA without PVA. The SPEA material exhibited a specific capacitance that was 1.4 times larger than that of the SPEA sample without PVA addition. Also, the water retention was improved using PVA. The internal architecture of the SPEA featured an outstanding three-dimensional pore network, through which conductive ions flowed to produce a force that countered stress and converted electrical energy into mechanical energy. Thus, strong conductivity and performance in actuation capabilities will also be reinforced in a definite ratio of doping.

Zheng et al. [109] investigated a PVA biopolymer membrane for a humidity-responsive and reprogrammable actuator, synthesized by grafting with 9-anthracenecarboxylic acid (9-AN), then blending with 2,2′,4,4′-tetrahydroxybenzophenone (THBP), followed by photo-crosslinking. The hydroxyl groups on the PVA allow quick moisture uptake and reversible swelling, making it an ideal candidate. Anthracene photodimerizes at 365 nm, where the illumination is applied, and THBP absorbs light across the thickness. This results in an irreversible crosslink density gradient, with more crosslinking on the irradiated side, and the internal and back surfaces being less crosslinked. When wet, PVA absorbs water and causes different levels of swelling due to the expansion of the loosely crosslinked region more than that of the closely crosslinked region; this causes a through-thickness mismatch strain, which leads to bending in the direction of the irradiation. The bending pattern can be reprogrammed: exposure to light shorter than 254 nm cleaves the anthracene dimers locally, and subsequent exposure to light longer than 365 nm rewrites it, allowing the local cleavage to be patterned with photomasks. At a constant humidity (RH = 20 to 90%), it attained the bending angle with an average velocity of 4.20°/s, followed by 2.53°/s and 0.92°/s. In a nonuniform humidity field of 182.20° of the bending angle at an average speed of 22.78°/s, full recovery was achieved in 92 s. The actuator remained stable over more than 10 cycles, demonstrating its durability. Significantly, the anthracene groups were UV responsive, thus providing programmable and reversible shape changes with selective crosslinking (365 nm UV) and cleavage (254 nm UV) to achieve complicated biomimetic applications such as a soft walking robot (rate 1.08 mm/min) and a flower-like actuator.

Similarly, Kumar et al. [110] have reported a dual-responsive soft actuator composed of a single layer of cassava starch and PEDOT:PSS, aiming to simplify fabrication and increase durability, as bilayer systems often experience the problem of delamination. The actuator demonstrated sensitivity to solvent vapors (water, acetone, and alcohol) as well as light (sunlight and infrared), exhibiting different directional deformations in response to each of the triggers. It also folded in opposite directions toward water vapors compared to alcohol vapors, and under light. Programmable bending direction was also supported by geometric surface patterns. The performance of the actuator was demonstrated by experimental forms in wearable fabrics, soft robotics, and smart gadgets.

Vahid Hasantabar and coworkers [111] demonstrated that a soft bio-electronic muscular actuator was designed on the flexible membrane of a double-network hydrogel (DNH), biopolymers of κ-Carrageenan (κ-Car) and PVA. To improve the membrane properties, the P(AA-co-AMPS), where acrylic acid (AA) and 2-Acrylamido-2-methylpropane sulfonic acid (AMPS) copolymers were grafted to κ-Car, which resulted in adding ionic groups (the -COOH and -SO_3_H) into the chains. The membrane was made functionally more capable of ionic conductivity, water uptake, and ion-exchange potential, and these functional groups did not compromise its biocompatibility and flexibility. When a low voltage is applied in the electrolyte, the mobile cations begin to migrate and charge the electric double layers at the hydrogel-electrode interfaces, creating an uneven distribution of ions and solvent across the thickness. The resulting osmotic pressure gradient causes an increase in the swelling on the cathodic side and a bending moment, which causes the strip to be turned away towards the anode. Carbon electrodes have high interfacial capacitance and flexibility that facilitate efficient charge storage and allow for maintaining repeatable curvature. The mechanical flexibility characteristic of PVA rendered it flexible enough to form a double-network together with κ-Car, which was further crosslinked both physically by KCl/ZrOCl_2_ and chemically by glutaraldehyde to provide an elastic, transparent, and strong membrane capable of performing actuator functions. The actuator was produced by placing the DNH free membrane between the two spray-coated/hot-pressed Vulcan carbon/functionalized multi-walled carbon nanotube (V/*f*-MWCNT) electrodes. The actuator with a membrane grafted with 50 wt% P(AA-co-AMPS)-κ-Car/PVA demonstrated the best performance, exhibiting a tip deflection of 49.3 mm, bending strains of 0.8%, water uptake of 58.8%, and a maximum ionic conductivity of 4.76 × 10^−5^ S/cm, a specific capacitance of 0.54 Mf/cm^2^, and a low charge transfer resistance of 2.39 × 10^3^ Ω.cm^2^. It also showed a low percentage of water loss (18%) and maintained its durability for 180 min of open exposure to air. These experimental findings are direct evidence of the success of combining κ-Car and PVA and using ionic grafting in creating green, low-cost yet high-performance pseudo-soft actuators.

In another study, a flexible ionic artificial muscle is introduced using sodium alginate-chitosan hydrogel with attention to the effect of the moisture content on the electromechanical properties [112]. With the systematic alteration of moisture levels in the electrolyte layer, the content of 78.64% moisture and the deflection displacement reached its highest value, 17.9 mm, and the maximum output force 5.99 mN at 10 V. They also carried out applications, which were exhibited by an example of a bionic flytrap, as a prototype of a biomimetic device with applications. Similarly, Yang et al. [113] fabricated a system with calcium alginate hydrogel as the flexible biomimetic artificial muscle (FBAM). The most optimal actuator attributes in the fabrication of the FBAM were attained when the calcium alginate gelling solution was used in a combination of CaH_8_I_2_O_4_ at a pH level of 5.34. The peak force density of 22.807 mN/g, operating life of 1066 s, the speed of response of 98 s, and the rise time of 0.1046 mN/gs. Nevertheless, the electrically actuated properties of FBAM were determined to be the most unfavorable when the gelation was performed with C_2_H_2_CaO_6_ with a pH of 9.08. This may be explained by the ionic migration mechanism that the calcium alginate reaction caused ionization of Ca^2+^ cations that are present in calcium salts, and this resulted in the replacement of H^+^ and Na^+^ cations present in the sodium alginate. The gelation process, thus, converted sodium alginate into calcium alginate hydrogel. The Ca^2+^ cations showed efficient performance at a pH of 5–6. The appropriate level of calcium alginate gelation led to the generation of inter-molecular bonds in calcium alginate hydrogel that contained polymer, β-D-mannuronic, and α-L-guluronic. The gelation process was inversely related to the pH level in the water medium of the electrically driven system membrane.

**Figure 5:**
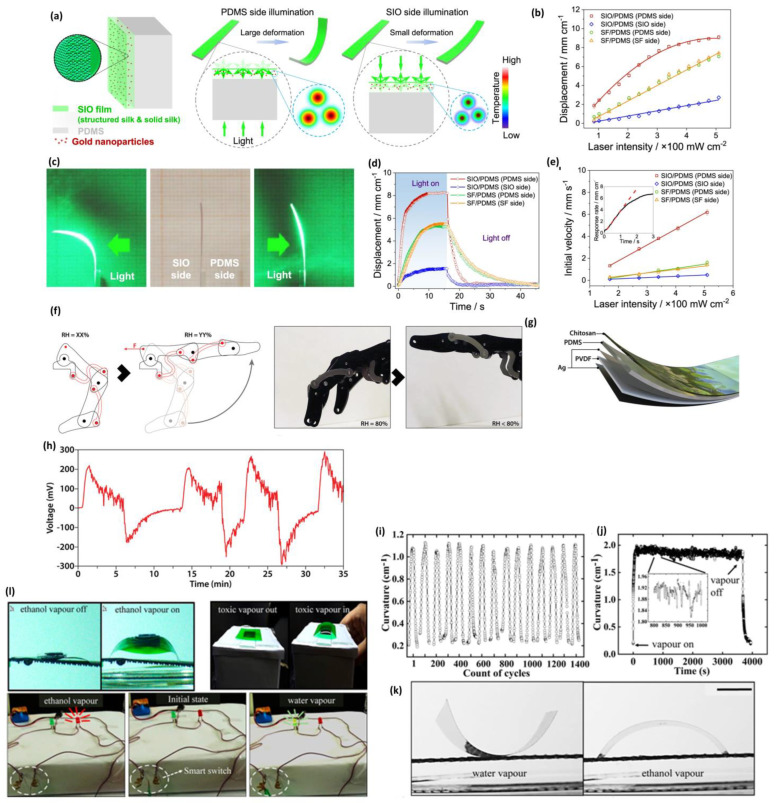
(**a**) Schematic representation of the photo-deformable bilayer film based on SIO and PDMS. Adapted with permission [114]. Copyright 2021 Nature Communications. (**b**–**e**) Photonic bilayer actuation behaviour. (**b**,**c**) Variation in the maximum displacement with laser power (**b**) and incident time (**c**). (**d**) Deformation of a photonic bilayer strip. (**e**) The rate of initial deformation of the bilayers depends on laser power. Adapted with permission [114]. Copyright 2021 Nature Communications. (**f**) Representation of the articulated finger, and pictures show four fingers at atmospheric humidity balance (**left**) and when they are dried (**right**). Adapted with permission [115]. Copyright 2023 Wiley-VCH GmbH. (**g**) Schematic representation of a multilayered piezoelectric film. Adapted with permission [115]. Copyright 2023 Wiley-VCH GmbH. (**h**) Output of the hydroelectric generator over drying/relaxing cycles. Adapted with permission [115]. Copyright 2023 Wiley-VCH GmbH. (**i**) The curve generated by the actuator is plotted at every 100th cycle. Adapted with permission [116]. Copyright 2024 American Chemical Society. (**j**) Varying the bending curvature of the starch film. Adapted with permission [116]. Copyright 2024 American Chemical Society. (**k**) Snapshots show opposing bending of starch film under water (**left**) and ethanol (**right**) vapors. Adapted with permission [116]. Copyright 2024 American Chemical Society. (**l**) Starch film lifts the load when exposed to ethanol vapor on the bottom side. (**l**, **top**) Application of the starch film load actuator to release vapor trapped within confined spaces. (**l**, **bottom**). Adapted with permission [116]. Copyright 2024 American Chemical Society.

Wang et al. [114] invented a biopolymer actuator when dominated with light, where the bimorph structure was designed in between the gold nanoparticle-doped silk fibroin inverse opal (SIO) as well as polydimethylsiloxane (PDMS) material. The SIO films were cast through the colloidal assembly of polystyrene nanospheres (300 nm) templates, filled with the regenerated silk fibroin solution that was heavily doped with 30 nm gold nanoparticles, and the removal of templates to achieve nanostructured photonic crystals. The bilayer was formed by spin-coating (~60 µm) PDMS (the thickness of the PDMS coating was not exceedingly precise) atop the SIO. When the SIO is illuminated, the AuNP-rich silk absorbs and localizes photothermal heating, causing a mismatch in thermal strain between the AuNP-rich silk and the PDMS, which results in bending. The photonic bandgap and its spatial patterning enable photonic heat deposition and curvature by tuning the photonic bandgap, as well as allowing shape morphing by programming and tracking the angles of the light. The reaction is fast, reversible, and constant with repeated cycles. This was attributed to the high sensitivity of the actuator to light since the negative coefficient of thermal expansion (CTE) of silk interacted with the high CTE material, PDMS, and bending is achieved by doping with light. The samples with PDMS illuminated on the side under a 532 nm laser (I = 35 mW/cm^2^) could produce the greatest displacement of greater than 20 mm of a 25 × 2 mm strip. They could be stably actuated over 100 cycles without failure. It was found that the response rate was maximum when it was illuminated on the PDMS side, thus indicating exponential bending kinetics. Figure 5a is relevant because it discusses the nanostructured silk inverse opal photonic lattice and how it helps to improve light-matter interaction. Additionally, Figure 5b–e reports on the actuator’s behaviors and displacement against laser intensity and cyclic performance, which states the efficiency in using the actuator. Figure 5c shows the actuator response at laser illumination (I = 35 m/cm^2^), and Figure 5d shows the deformation of a photonic bilayer strip (20 × 2 mm) by a green laser (I = 17 m/cm^2^). The inset is a calculation of the response rate, which is described as the slope of the tangent line. This allows considerable potential for silk-based biopolymer actuator applications in soft robotics, adaptive optical devices, and bio-integrated solar energy systems, as they are both biocompatible and can be tailored to tunable photonic properties. Through the application of reversible actuation mechanisms, they can be reconfigured for specific applications.

The biopolymers based on the use of chitin were shown to have a high potential in passive actuator applications due to their hygroscopicity and structural flexibility. In similar research, Rukmanikrishnan et al. [115] fabricated freestanding chitosan films (approximately 130.5 µm thick, 2 cm wide) made with shrimp-shell chitin that were designed to make use of water-powered molecular rearrangement to achieve mechanical work devoid of any external power. Hydroscopic actuation in freestanding chitosan films involves water absorption and release, which rearranges the hydrogen-bond network, leading to volumetric strain and causing macroscopic contraction or expansion without external energy. This response is conditioned by previous secondary reorientation (stress-induced alignment or crystallization), which reduces the available water sites and selectively directs the forces of hygroscopy, which can then produce large tensile responses during dehydration. The same water-exchange process is accelerated by chemical dehydration (e.g., ethanol). The forces generated by the films exceeded 45 N due to humidity changes and showed similar performance when driven by dehydration with ethanol, which more closely mimics biological movements. They proved to be effective as artificial muscles and were tested in a prototype mechanical hand that produced a force pull of 90 N per film; this allowed a grip strength of 18 kg. The fingers comprised three rigid segments and one pulling mechanism driven by a uniaxial force (see Figure 5f,g). All the films applied a force of 90 N, which gives a total grip capacity of around 18 kg, which is more than half the average grip strength, which is 30.6 kg in adults. Nonetheless, the potential of creating significant force with the help of biocompatible and thin chitinous films can be restricted by a hygroscopic mechanism, which is reliant on evaporation. The bending process was next translated into an electrical signal through the connection of a thin film piezoelectric device consisting of a 28 μm thick polyvinylidene fluoride (PVDF) membrane with silver metallization on both sides (Figure 5g). The hygroelectric generator was dried and equilibrated in turn with the air environment to mimic environmental modifications. This caused electric currents of hundreds of millivolts to be generated within several minutes for each of four different cycles (Figure 5h). Hygroscopic films were used as a hygroscopic energy harvester, and their response was used to move articulated fingers. The biocompatibility and sustainability of these entirely unmodified chitinous films, as well as their strong force output, have made them a promising biopolymer to be used in artificial muscles and actuation in biorobotics, as well as in self-powered bio systems.

Satapathy and co-workers [116] synthesized a relatively fast, reversible, and bidirectional actuator (moved nematically up and down at relatively high velocity) that was fully bio-degradable and made of cassava starch and was responsive to multivapors. Water vapor exposure enhances hydrogen-bonding and uptake at the irradiated surface, which destabilizes interchain hydrogen-bonds and leads to local expansion, which folds the film upwards. On the contrary, the bound water is extracted by ethanol (and other organic vapors), resulting in local contraction and bending down. The magnitude and sign of the bending is programmable by the water/ethanol ratio, and the bending axis is oriented by patterning the surface. The actuator was impressively durable, performing at least 1400 cycles of swelling and contraction with no loss of performance, since the bending curvature changed little in Figure 5i. Figure 5j demonstrates that it can also maintain a maximum curvature of approximately 1.9 cm^−1^ after around 3900 s of continuous exposure to water vapor with minimal fatigue, and the figure below is an inset photo that depicts a magnified fluctuation against time of 800–1000 s. It was also found that the bending behavior opposes the vapors, as shown in Figure 5k with a scale bar = 4 mm; when the starch film was subjected to water vapor, it bent upwards, and in ethanol, it bent downwards, which allows us to control the direction of the bending. The actuator exhibited impressive mechanical output. When exposed to ethanol vapor, it was able to lift 10 times its own weight (40 mg) and produce a force of 4.2 mN. On the left, the toxic solvent vapor is absent, with the window closed. On the right, the window is open, allowing vapor of toxins to escape or enter the room, as shown in Figure 5l, top. Working demonstrations of applications in flexible electronics showed its flexibility: a vapor-controlled safety window that opens and closes based on the presence of toxic vapors consisting of ethanol and DMF (Figure 5l, bottom), and a smart bidirectional electric switch which operates on the direction of bending of the film to switch red (on left) and green (on right) LEDs in the presence and absence of ethanol or water vapor. These results are indicative of the stability, sensitivity, and potential applications of the actuator as a soft robot, environmental sensing device, and biomedical device.

Recently, actuator technology has developed biopolymers as sustainable actuator platforms because of their inherent biocompatibility, flexibility, and biodegradability. To be used as actuators, biopolymers should have key features such as mechanical strength, including tensile strength, the ability to conduct ions, stimuli-responsive deformation, structural reversibility, and thermal and chemical stability. They should also respond to light, humidity, voltage, or vapor, and exhibit reversible deformations. The properties enable the biopolymer matrices to convert external stimuli into mechanical motion with the help of conductive fillers (e.g., MWCNTs, ionic liquids), responsive moieties (e.g., azobenzene, anthracene), and structural enhancers (e.g., PVA, silk fibroin). The examples reviewed in the literature demonstrate that the performance requirements, such as high bending angles, actuation speeds, low voltages, durability through cycles, and controllable responsiveness, are indeed met using biopolymer-based systems. This indicates that biopolymers can serve as alternatives to actuator materials without drawing a significant carbon footprint in fields such as soft robotics, bioelectronic devices, and environmental sensing platforms, among others.

## 5. Advances of Biodegradable Polymers in Healthcare Applications

Biopolymers have revolutionized healthcare research due to their high biocompatibility, controllable degradation, and tissue regenerative properties. These biopolymers can form a close affinity with the extracellular matrix of biological tissues, providing structural support and encouraging cellular differentiation, proliferation, and adhesion [117]. Their applications extend beyond wound healing, drug delivery, implantable medical devices, and orthopedic devices [118]. For example, PLA composites that, when reinforced with hydroxyapatite [119] or bioactive glass [120], have registered improvements in tensile strength and bioactivity. Similarly, polycaprolactone with hydroxyapatite has demonstrated a better scaffold for tissue engineering [121]. Advanced processing techniques, such as additive manufacturing and surface coating, have led to the development of biodegradable systems with tunable properties. These systems are now opening up new frontiers in sustainable healthcare solutions.

Recently, chitosan-modified liposomes were comprehensively discussed as a possible way of delivering the natural anti-tumor agent curcumin (CUR). Wan et al. [122] designs a platelet-loaded membrane-inspired chitosan-modified liposome (PCLP-CUR) to selectively treat cancer based on the earlier study of platelet membrane-camouflaged nanoparticles. The system exhibits a mean hydrodynamic diameter of 162.8 nm, an encapsulation efficiency of 91.69% and a change in surface charge between −43.7 mV (LP-CUR) to +42.0 mV with chitosan modification and then to +22.2 mV after platelet membrane fusion. This was stable for 10 days at 4 °C, and encapsulation is retained. It depicted that the biomimetic PCLP-CUR lipidic nano carriers exhibit high biocompatibility, higher bioavailability, prolonged blood circulation, and enhanced tumor specificity. It is fascinating how the addition of chitosan improved the drug release behavior and enabled cargo to be released quickly under tumor conditions, which occur in a slightly acidic environment. Hence, the drug release has pH-responsive properties over 24 h, ~60% at pH 5.0, 49% at pH 6.5, and 34% at pH 7.4, enabling endosomal and lysosomal release with a minimum release at neutral pH. PCLP-CUR’s pH responsiveness enables quicker drug release in acidic environments, which could help minimize chemotherapy’s harmful effects on healthy cells. The next key finding was that PCLP appeared not to be absorbed by macrophages, probably because of the protein CD47 present on the platelet membrane. In addition, the in vitro and in vivo experiments underpinned the enhanced tumor targeting ability of PCLP-CUR. This can be attributed to the fact that P-selectin on the platelet membrane binds with the CD44 receptor on Hep G2 cells. In vitro, in vivo (using tumor-bearing Hep G2 mice), the anti-cancerous activity of PCLP-CUR is found to be higher than that of other CUR derivatives, and PCLP-CUR treatment produces tremendous shrinkage of the tumor. In rats, pharmacokinetic analysis indicates PCLP-CUR area under the concentration-time curve is 336.99 µg/L.h, which is 2.51 times higher than that of free curcumin and 1.44 times higher than that of conventional liposomal curcumin, with an increase in mean residence time of 3.60 times compared to free drug and 2.15 times compared to LP-CUR. The modification of the chitosan could accelerate the drug release in the tumor sites. The in vitro biocompatibility is supported by no apparent cytotoxicity of PCLP to either HUVECs or HepG2 cells up to 800 μg/mL (48 h) and by continued weight gain and no histopathological organ damage on H&E staining in vivo. Bioactivity is evidenced by high tumor-growth inhibition compared to free CUR and LP-CUR, observable through increased TUNEL-positive and reduced Ki-67-positive tumor cells, as well as supporting the active tumor-targeting ability, and not proliferation by platelet membrane. This is probably due to PCLP-CUR’s use of natural polysaccharides and biocompatible cell membranes.

Yuan et al. [123] synthesized natural polysaccharides, Bletilla striata polysaccharide (BSP) and chitosan (CS) blend and bilayer membranes with excellent biocompatibility, biodegradability, and film-forming properties. The interactions and network structure provide mechanical reinforcement in BSP/chitosan (BSP/CS) membranes. Chitosan cationic chains create strong hydrogen bonds with BSP, which has numerous hydroxyl and carboxyl groups. This enhances the adhesion between chains as well as the effective crosslink density. The highest performance is observed at optimal composition (75/25 BSP/CS), where the most efficient load transfer with tensile strength 108.23 ± 0.3 MPa and elongation at break 44.35 ± 0.6%, which is higher than that of the neat chitosan membrane. BSP has rich hydroxyl groups, thus directly suppressing radical species. These groups can eliminate the 1, 1-diphenyl-2-picrylhydrazyl (DPPH) in the DPPH assay by reducing DPPH to DPPH-H to produce the measurable reduction in absorbance; in the hydroxyl radical assay, they can react competitively with OH through hydrogen transfer. Based on this process, scavenging efficiency rises systematically as one adds more BSP to the system, with the 75/25 BSP/CS film attaining 35.56% DPPH quenching. The membranes reduced the accumulation of reactive oxygen and, therefore, promoted wound healing. The ability to scavenge hydroxyl radicals was also increased with BSP. This is due to the hydrophilic nature of the carbohydrate-based chemistry of BSP and chitosan, which facilitates benign protein adsorption and reduces membrane damage that can occur due to nonspecific adsorption. Non-cytotoxic characteristics of in vitro cytocompatibility testing (CCK-8) were observed in all extract dilutions, and fibroblast viability remained above 80%. The membranes could inhibit *E. coli*, P. aeruginosa, and S. aureus bacterial strains, mainly due to the cationic interaction of chitosan with bacterial membranes. As shown by the cytotoxicity tests on L-929 fibroblasts at all levels of non-toxicity (more than 80% viable cell culture), it demonstrated high biocompatibility, which is essential for wound healing materials. The application of chitosan improves antibacterial performance, as the protonated -NH_3_^+^ groups electrostatically interact with the negatively charged bacterial colonies, promoting an increase in membrane permeability. The colony-forming units were reduced to near control levels on high-CS and bilayer constructs (i.e., *S. aureus* 330 ± 20.6 CFU control versus approximately 1 CFU on CS/bilayer), thereby demonstrating significant bacteriostatic activity. Furthermore, enzymatic biodegradation under lysozyme was capable of being tailored by composition, with cumulative mass loss after the testing period amounting to 50.8 ± 3.4%, 44.7 ± 1.1%, 39.7 ± 0.7%, and 24.0 ± 6.9%, as the BSP content increased, with the bilayer exhibiting the highest degradation at 47.8 ± 2.1%.

In a different study [124] nanofibrous membranes of electrospun fibres by embedding the eucalyptus oil in nano-chitosan particles and then adding it to cellulose acetate (CA), creating a nanocomposite membrane for wound dressing. CA works as a matrix, and the combination of protonated chitosan and eucalyptus oil synergistically disrupts microbial envelopes, reducing the number of viable microbes. This is evident from the large zones of inhibition and rapid time-kill kinetics observed against S. aureus. Nanoparticles formed showed a size of 48.26–58.24 nm on average, a zeta potential of +19 mV, and a high encapsulation efficiency of 92.4%. With an elastic stress of around 1.52 MPa, an elastic modulus of approximately 35 MPa, an elastic strain of about 4%, and a breaking strain of roughly 8.8%, the membrane nanofibers show good mechanical integrity. The nano-chitosan/eucalyptus-oil/CA membranes are characterized by homogenous fibers of about 98 nm in diameter, and hence uniform load transfer. The nanofibers have confirmed sustained release of eucalyptus oil within 24 h, and they remained highly mechanically stable. The antimicrobial efficacy demonstrated an inhibition zone of 43.0 ± 6.0 mm against *S. aureus*. The incorporation of agar diffusion reductions (R%) was 78.0 ± 5.0% for *P. vulgaris*, 65.0 ± 6.1% for *C. albicans*, and 87.0 ± 2.4% for S. aureus when integrated into cellulose acetate (CA) nanofibers. Clear zones measured approximately 42.0 ± 1.0 mm, 30.0 ± 1.0 mm, and 57.0 ± 3.0 mm, respectively. Cytocompatibility assays with human melanocytes (HFB4) indicated satisfactory cell viability at the tested concentrations, with 80% viability at 1.5 mg/mL of nano-chitosan/eucalyptus oil/CA and 78% viability at 1.5 mg/mL of eucalyptus oil/CA. Cell viability decreased to approximately 60% at a high dose of 12.5 mg/mL, establishing the viable dose range for biomedical applications. The mechanistic investigations showed an increase in TGF-β and type I and type III collagen expression, which is necessary to repair a wound.

Ahmadi et al. [125] designed controlled release capability of chitosan/gelatin nanofiber membranes that they use to inhibit the occurrence of infection by adding cinnamon extract (CE) at the site of implantation. They explored the fact that the addition of the extract to the nanofibers prompted an improved degradation characteristic, biocompatibility, and antimicrobial properties. The CE-loaded electrospun chitosan/gelatin (Chi/Gel) membrane is a physically entrapped nanofibrous network formed by glutaraldehyde crosslinking. Consequently, it releases into solution in a two-stage profile, which forms the basis of the bioactivity, an initial burst release over the first 6 h, followed by a near-zero-order release thereafter, with about 70% of the compound being released cumulatively during the first 6 days (projected to be about 16 days overall). The cells were grown and attached to the nanofiber membrane, proliferating at a high cell viability capacity until the CE content reached 4%. The electrospun fibers exhibit uniformity in their structural composition, with their mean diameters recorded as 168 ± 75 nm (0% CE), 165 ± 69 nm (2%), 166 ± 50 nm (4%), and 141 ± 37 nm (6%). This phenomenon may occur because the CE possesses low polarity and does not bear any electric charge; therefore, it does not affect the conductivity of the polymer solution. Crosslinking results in an elevation of the water contact angle, approximately 38 ± 4°, while the high-loaded mats containing CE exhibit contact angles near 80 ± 3°. Conversely, the contact angle of high-loaded mats with CE at 4% and 6% is approximately 80 ± 2°. The antibacterial efficacy shows a dose-dependent relationship, reaching its peak at 4% CE, where viable colonies against S. aureus decrease to 372 ± 40 CFU/mL with a corresponding activity of 90 ± 6%, and against *E. coli* to 670 ± 33 CFU/mL with 82 ± 5% activity (controls approximately 3722–3723 CFU/mL). Cytocompatibility, assessed by fibroblast MTT assay, significantly improves at 2% and 4% CE compared to the control on 7 days (*p* < 0.01). Conversely, 6% CE reduces cell viability, aligning with known limitations of cinnamaldehyde dosage. Scanning electron microscopy confirms strong cell attachment and spreads on mats containing 4% CE. Another group [126] investigated silver nanoparticles (AgNPs) integration into chitosan-based membranes for antibacterial and wound healing ability. The bioactivity of the membrane in phosphate-buffered saline (PBS) showed inhibition of S. aureus, which lasted up to 28 days. Nonetheless, after pre-immersion in Fetal Bovine Serum (FBS), only the Ag5 sample showed a temporary zone of inhibition that was observed up to 4 days. In vivo experiments demonstrated that high loading membranes, had the effect of eliminating subcutaneous S. aureus infections to levels that were undetectable in most implants by 28 days and that sterilization correlated with the amount of silver (Ag) more than 250 ng per capsule. On the other hand, it did not significantly decrease the bacteria in the lower loading membranes. Biocompatibility studies provided evidence of normal wound healing and full-thickness wounds reduced on 14 days, with no statistically significant differences appearing between collagen deposition, neovascularization, or macrophage density. In addition, Ag-impregnated dressings were likely to help with faster macroscopic wound reduction compared to sham controls. Chitosan provides stiffness, while polyethylene oxide offers ductility, resulting in greater tensile strength and continued elongation. Well-dispersed in situ-formed AgNPs act as reinforcements; however, high levels of Ag induce defects that decrease strength. The mechanical testing showed that the membranes were not too rigid, and the elongation at break was about 6 to 8%. It is important to note that the membranes were the strongest and stiffest with the Young’s modulus (841 ± 165 MPa) and tensile strength (22.21 ± 5.03 MPa).

Tissue regeneration using bio-membrane/scaffold is becoming a critical application in the medical world, particularly as a periodontal application. A blend of carrageenan and PVA was designed by Sudhakar et al. [127] about the physical and mechanical properties of the blend. The biopolymer carrageenan, together with PVA crosslinked with 3-aminopropyltriethoxysilane (APTES), was produced in the scaffold/bio-membrane in this study. This membrane strength of 89.21 MPa was revealed to be significantly superior to that of commercial membranes. The membrane surface is smooth, which encourages the adhesion and proliferation of cells. In addition to this, the carrageenan bio-membrane promotes the growth of fibroblast cells, which are favorable in restoring dental tissues. Therefore, the blend of carrageenan biopolymer and PVA has been determined as suitable for the formation of a regenerative polymeric biomaterial to be used in dental and other biomedical purposes. Recently, Patel et al. [128] generated CNT-coated biopolymer nanofibers with carefully chosen bi-modal nanoscale topographic characteristics that regulated key biological responses in vivo. The CNT functionalization was able to significantly suppress inflammatory activity and minimize macrophage accumulation at the site of implantation. In vivo, bone regeneration was greatly improved by increased bone mineral density and the elevation of osteogenesis-related genes, including osteopontin (OPN), osteocalcin (OCN), and bone morphogenetic protein-2 (BMP2). In vitro studies, in which mesenchymal stem cells (hMSCs) were exposed to CNT-modified nanofibers with coded surfaces, provided evidence of rapid cellular adhesion, osteogenic differentiation, and mineralization on CNT-coated nanofiber surfaces. MSC osteogenesis protein BSP and OCN were up-regulated by about 40–50-fold relative to CNT0 (pure PCL) at 14 days, and mineralization of the matrix was enhanced by about threefold relative to CNT0 on 28 days, suggesting a high process of promoting mineralization. The cellular bioactivity was also confirmed through the quick development of apatite, whereby specimens were immersed for 7–28 days, which was confirmed through the ATR-FTIR and the XRD tests. The peri-implant fibrous capsule, which was about 40–50 µm in CNT0 and 10–20 µm in CNT1, in vivo, is associated with the increased pro-healing responses that can support the bone regeneration ability of the scaffold. Overall, these results suggest the potential of CNT-engineered biopolymer scaffolds in regenerative applications, especially in bone tissue engineering, where it is essential to modulate inflammation and vascularization together with osteogenesis. A thin CNT mesh-like coating onto alkaline-activated PCL nanofibers maintained the final tensile strength as compared to uncoated PCL. Still, it yielded a minor decrease in elastic modulus, with corresponding increases in elongation and energy dissipation, which is desired in applications where toughness and damage tolerance are key factors in addition to tensile strength. Alkaline treatment creates surface -OH/-COOH groups, enabling strong electrostatic and hydrogen bonds with charged CNTs (42.3 mV).

Lin et al. [129] introduce the fabrication of pH-sensitive hydrophobic/hydrophilic antibacterial fibrous films made up of biodegradable polyhydroxybutyrate (PHB) and PCL. The functionalization of the membranes was carried out by electrospinning and in situ polymerization with N-halamine quaternary ammonium salt (HQAS) compounds to act as antimicrobial agents and with dimethylaminoethyl methacrylate (DMAEMA) to demonstrate pH-responsive wettability. After treatment with HCl, it was found that the membrane was hydrophilic and exhibited a good antibacterial property (*S. aureus *reduction was found to be 100% in 30 min and 72.4% of *E. coli* in 60 min). Its thrombogenic property had improved (the blood clotting index was found to be 60.6%). Conversely, membranes treated with NH_4_OH proved to be hydrophobic with a contact angle of 134° and had antifouling effects; thus, the bacterial adhesion was reduced. The membranes showed high thermal stability, reusability by rechlorination, as well as UVA/dark storage stability. They also confirmed cytocompatibility tests in fibroblast cells. The other attractive work on a bioinspired approach to bio-fabrication of nanofibrous composite scaffolds [130] through uniform dispersion of chitin nanofibrils (CNF) in PCL membranes as a new biomedical material. CNF is a hydrophilic natural polymer, which was obtained by acid hydrolysis of chitin and dispersed in trifluoroethanol. Nanofiber meshes and films were prepared by electrospinning and spin-coating, with varying ratios of PCL and CNF (100/0 to 25/75). In vitro experiments in NIH-3T3 fibroblasts showed outstanding cell activity and attachment to all compositions. Furthermore, the ability of the composite, specifically 75/25 PCL/CNF, to facilitate the process of tight junction formation during endothelial cell (HUVEC) culturing and transendothelial electrical resistance (TEER) measurements indicated that it could be an appropriate choice of material to be used in blood–brain barrier models. Researchers examine the development of starch-based biodegradable foams as scaffold materials in neural tissue engineering since there is an urgent need to have 3D biocompatible scaffolds that mimic such properties of the tissue. Wen et al. [131] have devised a rapid foaming technique that can immobilize live cells within the porous starch matrix and exhibit high cell viability. Neural markers, such as the βIII-tubulin marker, were highly expressed through immunostaining, indicating successful neural differentiation and an active cell–material interface. The research proposes a cost-effective, scalable method that enables the generation of 3D cell-loaded constructs to deal with neural tissue repair, organoid creation, and advanced neurobiomedical applications. Similarly, the effect of starch on electrospun polyhydroxybutyrate (PHB) scaffolds for tissue engineering was investigated by Asl et al. [132]. Addition of starch increased the hydrophilicity, biodegradation, and cell viability of the scaffolds. Proliferation and viability of MG-63 cells and alkaline phosphatase (ALP) activity increased significantly in PHB-starch scaffolds.

**Figure 6:**
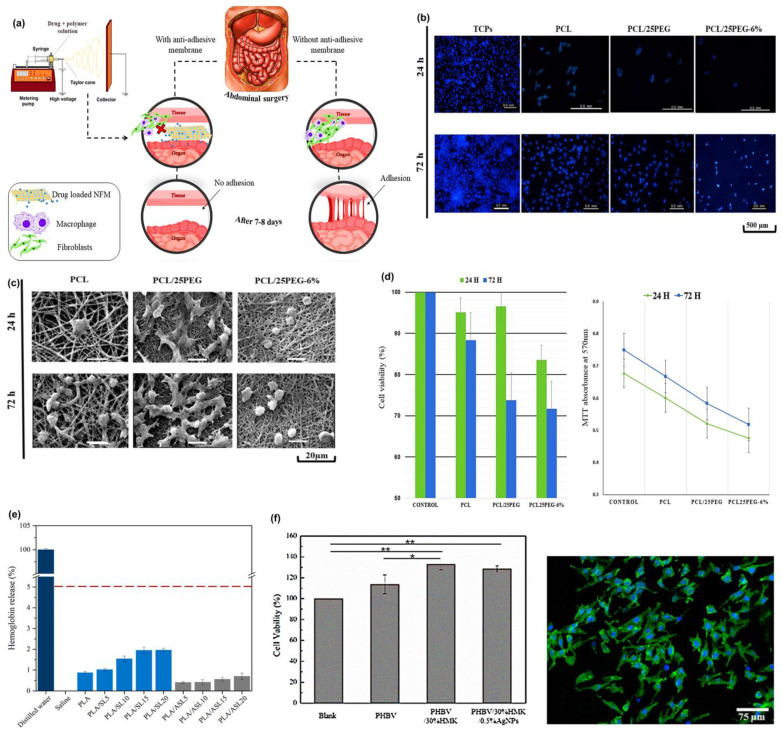
(**a**) Schematic representation of the adhesion process and the importance of the antiadhesive barrier. Adapted with permission [133]. Copyright 2022 American Chemical Society. (**b**) DAPI-stained L-929 fibroblasts observed after 24 and 72 h of seeding, scale bar = 500 µm. Adapted with permission [133]. Copyright 2022 American Chemical Society. (**c**) SEM images of L-929 fibroblasts after seeding on days 1 and 3, scale bar = 20 µm. Adapted with permission [133]. Copyright 2022 American Chemical Society. (**d**) Number of fibroblasts adhered to the nanofiber surface at 24 and 72 h of cell culture. Adapted with permission [133]. Copyright 2022 American Chemical Society. (**e**) Release of Hemoglobin (%) PLA, PLA/SL, and PLA/ASL composites. Adapted with permission [135]. Copyright 2023 American Chemical Society. (**f**) (**left**) Viability of L929 cells on PHBV, PHBV/30% HMK, and PHBV/30% HMK/0.5% AgNPs nanofibrous mats. (**right**) confocal laser scanning microscopy image of cells from L929 growing on the PHBV/30%HMK/0.5%AgNPs nanofibrous scaffold mats, scale bar = 75 µm. Adapted with permission [137]. Copyright 2020 Elsevier.

Kheilnezhad and Hadjizadeh [133] demonstrate the efficacy of electrospun nanofibrous membranes loaded with ibuprofen in preventing postoperative abdominal adhesions, as illustrated in Figure 6a, using a biopolymeric blend of PCL and polyethylene glycol (PEG). The PCL/25PEG-6%, membrane had the best properties, such as the decreased diameter of fibers (~447 nm); decreased pore size (of <2 µm); high porosity (of ~85.8%); moderate hydrophilicity (water contact angle of ~75°), vital to active antiadhesion task. The presence of ibuprofen provided a sustained release (approximately 80% in 14 days), which helps in achieving localized anti-inflammatory effects through the inhibition of cyclooxygenase pathways, as well as the inhibition of fibroblast proliferation. The combination of PEG and ibuprofen resulted in improved solubility of the drug, as well as enhanced wettability of the membrane surface, making the drug more biocompatible and functional. There is an anti-adhesive effect of the ibuprofen-loaded biopolymer membrane, as shown in Figure 6b. It is concluded that the low density of the cells and their nuclear morphology are characterized by a spindle shape of the nuclei on the PCL/25PEG-6% membrane support that slows the release of ibuprofen, prevents the attachment and spreading of fibroblasts successfully, and, therefore, the membrane could be incorporated as a physical and pharmacological barrier to stop the formation of postoperative abdominal adhesions. SEM images of L-929 cell fibroblasts grown on days 1 and 3 are shown in Figure 6c. The fibroblasts are spindle-shaped and elongated, and they show successful adhesion, spreading, as well as potential proliferation with time. Conversely, the fibroblasts maintained a rounded and spindle shape, especially on PCL/25PEG-6% membrane (containing ibuprofen), even after 3 days. The cell density is also decreased in comparison with that of the drug-free membranes. Biological tests proved the decreased fibroblast adhesion and proliferation reflected by the reduced amount of DAPI-stained cells and the circular structure of nuclei in SEM images, as well as the cell viability over 70% at 72 h, implying the low level of cytotoxicity. Figure 6d illustrates bar graphs showing the percentage of cell viability and cytotoxicity of cells expressing fibroblasts after 24 and 72 h incubation on PCL, PCL/25PEG, and PCL/25PEG-6% membranes. The membrane that resulted in the lowest cell viability was the PCL/25PEG-6% with an approximate 83% cell viability at 24 h and 71% at 72 h. This was due to the membrane being soaked in ibuprofen, which can inhibit the proliferation of fibroblasts, an effect necessary for antiadhesive applications. In a recent study, Gabriel et al. [134] investigated an electrospun scaffold based on PCL blended with starch, reinforced with CaO, for tissue engineering. The scaffolds exhibited favourable biological behaviour, permitting cell adhesion and proliferation of M3T3-E1 preosteoblastic cells. In vivo experiments were conducted in Wistar rats using a subdermal dorsal model and resulted in enhanced biocompatibility and subsequent enhanced resorption process as compared to the pure PCL matrix. This biological study indicated that the PCL/Starch/CaO electrospun tissues may be appropriate scaffolds in guiding the bone tissue to regenerate.

Mearaj et al. [135] addressed the bioactivity and hemocompatible PLA-lignin biocomposites that can be utilized in the biomedical field and especially in antioxidant activities. PLA was incorporated with either raw soda lignin (SL) or acetylated soda lignin (ASL) in order to enhance the mechanical properties, antioxidant behavior and cytocompatibility. With the addition of lignin, the UV-blocking effect of PLA, surface hydrophobicity, and radical scavenging property were increased. The PLA/ASL5 acetylated-lignin composite showed the highest overall performance with tensile strength of about 57 MPa and a break elongation of about 10%. Neat PLA, on the other hand, showed a brittle nature with a low elongation of only approximately 5%. In most cases, the tensile strength of PLA/lignin composite was lower with the increase in the lignin loading, and is about 38 MPa at 20 wt% of lignin. The PLA/ASL composites were found to be better than the PLA/SL composites in strength and ductility in all loadings. This is due to the fact that the acetylation increases interfacial compatibility through the acetylation, which is viewed as an internal plasticizer. The antioxidant properties of PLA/SL composites were also good, and radical scavenging ranged up to 86.1% in the case of PLA/SL20. These composites also had a cell proliferative and antioxidant potential in human colon (HCT-15) and gastric (NCC-24) cell lines. Although ASL enhanced the mechanical qualities attributed to improved interaction with PLA, it exhibited low antioxidant activity due to the hindrance of the phenolic hydroxyl groups. As Figure 6e shows, PLA lignin composites are safe and hemocompatible, and can be used in biomedical devices that come in contact with blood directly. Hemolysis of all PLA/SL and PLA/ASL composites was <2%, which was compliant with the requirement of ISO 10993-4 [136], which specifies non-hemolytic materials. The hemolysis of PLA/ASL composites was even less than that of PLA/SL, not only due to increased hydrophobicity (demonstrated by higher values of the water contact angle) of the former ones. Jin-Peng et al. [137] prove that nanofibrous membranes with high molecular weight keratin (HMK) can be developed to have potential aspects of use in biomedicine, including wound healing. Keratinase was used to enzymatically extract HMK from wool and crosslink it with transglutaminase (TGase) to increase both the molecular weight and mechanical properties. The HMK was then co-electrospun with PHBV polymer and silver nanoparticles (AgNPs) introduced through in situ bioreduction in order to provide antibacterial activity. PHBV mats have a Young’s modulus of 94.6 ± 0.6 MPa, and the addition of 30 wt% of HMK gives a 43.4 ± 1.9 MPa (compared to 30.8 ± 0.3 MPa with uncrosslinked keratin) and tensile strength of 3.2 ± 0.1 MPa with transglutaminase crosslinking. In vitro, neat PHBV does not lose more than 7% of its mass after 28 days in PBS, whereas PHBV/30% HMK loses roughly 42.6% in the same time period, indicating that degradation is also controllable by keratin content. In vivo experiments demonstrated faster wound healing, and, thus, these membranes would be helpful in tissue engineering and regenerative medicine. Biocompatibility of the fabricated membranes is shown in Figure 6f, left. The cell viability of L929 fibroblast cells grown on PHBV/30%HMK and PHBV/30%HMK/0.5%AgNPs mats was significantly higher (~133% and ~128%, respectively) than that of the control, which indicated non-cytotoxicity and greater cell proliferation. Confocal microscopy images (Figure 6f, right side) showed that cells could adhere and spread effectively, exhibiting long filopodia and lamellipodia. This demonstrates that the nanofibrous structure based on keratin facilitates healthy cell attachment and growth, making it suitable for wound healing purposes.

One of the major challenges in delivering drugs to the colon is achieving effective colon-targeted drug delivery to treat conditions like Crohn’s disease in a highly variable pH environment as the medication transits through the gastrointestinal tract. Ayub et al. [138] conducted a study that led to the introduction of a new approach towards enhancing the targeting of cancer cell types to deliver Paclitaxel. They have designed biodegradable nanoparticles out of thiolated sodium alginate with cysteamine-based disulfide bridges through layer-by-layer self-assembly method. The oxidation of sodium alginate was performed using sodium periodate, after which it could be treated with cysteamine hydrochloride to introduce disulfide bridges. Such covalent crosslinks blocked the early release of drugs. The derived nanocarrier system exhibited a high encapsulation efficiency of 77.1% and sustained release characteristics. In vitro studies with HT-29 human colon adenocarcinoma cells have demonstrated excellent biocompatibility, with cell viability of 86.7%. These observations provide evidence of the potential of this system to deliver colon-specific drugs with a decreased risk of off-target toxicity.

Interestingly, HV@BC@TBG, an engineered living artificial skin, is proposed by Liu et al. [139] that imitates the multifunctional natural skin to accelerate the healing of chronic diabetic wounds. Bacterial cellulose (BC) was selected as a biopolymer scaffold because of its high biocompatibility, hygroscopicity, low cost, and simple microbial production. However, BC naturally lacks any antibacterial or bioactive properties. Hence, the researchers designed each side of the BC matrix with different functionalities to address chronic wound issues, including bacterial infection and impaired microcirculation. The outer layer was modified with a glucose-derivatized photosensitizer (TBG) that can produce reactive oxygen species upon light activation, thereby sterilizing the wound area. At the same time, a layer of vascular endothelial growth factor (VEGF)-transfected human umbilical vein endothelial cells (HV) near the wound was grafted by bio-orthogonal reaction, and continuous VEGF release occurred to induce angiogenesis and stimulate the proliferation of fibroblasts. The preparation process involved a layer-by-layer static culture of BC-producing K. sucrofermentans using sequential carbon substrates, resulting in a ~25 μm thick dual-functional BC film within 7 days. Grafting of VEGF-expressing cells on the layer of azide-rich material completed the assembly of the living artificial skin. They exhibited a high degree of antibacterial activity in the presence of light on the TBG side and the ability to release therapeutic levels of VEGF on the HV side, resulting in a synergistic effect causing faster wound healing and tissue regeneration on the induction of diabetic animal models. Artificial skin (N_3_@BC@TBG) and BC show an ultimate tensile strength of 9.57 MPa in contrast to 6.3 MPa of neat bacterial cellulose, and indicate enhanced strength and ductility without any change to the nanofibrous BC structure; thermogravimetric analysis also confirms the behavior of type I-β cellulose with rapid pyrolysis of 280–400 °C. This dual-functional system was able to simultaneously treat chronic wound healing but also exhibited good biosafety and translation potential of next-generation regenerative medicine, with wider applications in drug delivery, cytokine therapy, and tissue engineering. An endotoxin-free Escherichia coli strain is engineered by Pounraj et al. [140] to synthesize polyhydroxybutyrate (PHB) biopolymer particles (BPs) and uniformly display tumor neoantigen peptide (gp70, MHC-I and MHC-II epitopes) by fusing them with PHB synthase, which folds itself on the particle surface. The BPs of negative charge are readily captured by the dendritic cells, allowing antigen processing and subsequent presentation of MHC I/II, which stimulates T cells. Vaccination of a 4T1 metastatic mouse model of breast cancer with gp70-BP slowed tumor growth, reduced lung metastases, and altered the tumor microenvironment by enhancing infiltration of CD4+/CD8+ T-cells, decreasing lymphocyte attenuator BTLA (exhaustion marker), and increasing the facade of death ligand FasL on tumor-infiltrating lymphocytes TILs), as expected with Fas-mediated tumor apoptosis. The IL-12, IFN-g, and IL-23 serum levels increased, indicating a Th1-biased response. This platform also showed synergy with anti-PD-1, which inhibited further tumor growth. Mechanistically, particles act as a biodegradable PHB core (degrading to 3-hydroxybutyrate) biopolymer vaccine depot and delivery scaffold, concentrating neoantigens, enhancing APC uptake, and inducing a neoantigen-specific cellular response. This response suppresses primary tumor growth and metastasis. This study makes neoantigen-coated PHB BPs a versatile, scalable cancer immunotherapy platform that could be used to personalize immunotherapy. Hung et al. [141] produce biopolymer/gold nanocomposites by entrapment of gold nanoparticles into fibronectin (FN) and type I collagen (Col) matrices to produce mimicking extracellular matrix FN-Au and Col-Au surfaces. The materials exhibit nano-topography that is tuned and FTIR/UV-Vis signatures that indicate a protein-Au interaction, which subsequently alters cell behavior. On the mechanistic level, the AuNP-ECM hybrid enhances integrin α2, α4, and αVβ3 signals, MMP-2/9 (facilitates migration and remodeling), intracellular ROS, platelet activation, and monocyte-macrophage conversion, and alters the cell-cycle distribution of mesenchymal stem cells (MSC) towards the S phase with lower levels of pro-apoptotic proteins. They prepare tissue engineering and vascular graft surfaces, as well as provide a general surface modification for implants. In a one-month study involving subcutaneous rat implantation, the fibrous capsules were significantly thinner, and collagen deposition was lower around FN-Au and Col-Au compared to control proteins. The study also found a pro-healing immune profile, including lower levels of CD86/M1, higher levels of CD163/M2, and increased levels of CD31 staining, which indicates endothelialization. These in vivo and in vitro results on the antithrombogenic and anti-inflammatory effects of ECM/Au nanocomposites support the long-term compatibility of this nanomaterial and its potential as a durable, pro-regenerative interface for biomedical devices.

Controlling key functional properties of biopolymers allows for their use in designing effective materials, which find applications in tissue engineering, wound healing, antibacterial dressings, and drug delivery. They exhibit high biocompatibility, biodegradability, mechanical strength, and moisture retention, as well as responsiveness to environmental factors such as pH or temperature. Low cytotoxicity, combined with antimicrobial activity, is also crucial in preventing infections and ensuring the safe growth of cells. The studies discussed have proven to be highly effective in achieving these objectives. Specifically, PCLP-CUR was produced by modifying chitosan to form platelet-mimicking liposomes, thereby achieving targeted curcumin carrier delivery based on pH response and enhancing therapeutic effects. Likewise, the BSP/chitosan membranes also enhanced the mechanical properties up to 108 MPa, antioxidant activity (35.56% DPPH scavenging), and antibacterial action, while maintaining cell viability above 80%. Electrospun chitosan/eucalyptus oil-cellulose acetate composites resulted in an 87% decrease in bacterial growth and promoted tissue regeneration. Anti-adhesive PCL/PEG-ibuprofen membranes exhibited a sustained drug release and inhibited fibroblast proliferation. PLA-lignin composites exhibited enhanced antioxidant properties (up to 86%) and retained their hemocompatibility (<2% hemolysis), while keratin-based nanofibers improved wound healing and cell attachment. These systems achieved the desired performance thresholds, demonstrating that biopolymers can serve as highly functional platforms for next-generation biomedical devices when carefully engineered and designed.

## 6. Conclusions and Future Perspectives

**Figure 7:**
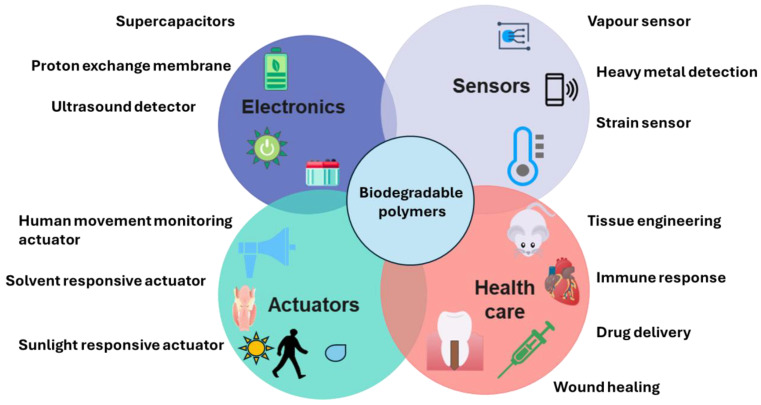
An overall schematic representation of biodegradable polymers for their application in electronics, sensors, actuators, and healthcare.

With growing attention for renewable energy, biopolymers are multifunctional alternatives for next-generation materials used beyond packaging, especially in flexible electronics, wearable sensors, soft actuators, and biomedical devices a schematic overview is shown in Figure 7. Their biocompatibility, biodegradability, and bio-functionality make them attractive for high-end applications. Shifting focus toward biopolymers rather than traditional polymers may help mitigate the growing environmental burden of electronic waste. The combination of compositional tuning, hierarchical assembly, and the incorporation of functional nanomaterials into biopolymers offers targeted performance that exceeds that of traditional synthetic polymers in both conventional and industrial applications.

This review highlights the various applications of biodegradable electronics, which include proton exchange membranes, flexible photovoltaics, piezoelectric sensors, electrolyte membranes, and supercapacitors. Structural modifications and nanocomposites strategies, including crosslinking chitosan with sulfonic acids, embedding silica or sulfonated nanoparticles, and incorporating protic ionic liquids, have resulted in significant improvements in proton conductivity up to 7.82 × 10^−2^ S/cm, water uptake, and electrochemical stability. Remarkably, chitosan devices achieved energy densities of 4.39 Wh/kg in supercapacitors and exhibit high piezoelectric sensitivity (up to 80 mV/kPa) in flexion ultrasonic transducers. Separators and active electrodes in energy storage devices have also been made using biopolymers such as PLA, cellulose, and alginate, which exhibit a superior specific capacitance of 233.6 F/g when PLA-derived carbons are used and show excellent cycling stability. Sensors with high selectivity, low detection limits with 2.94 nM for Fe^3+^, 3.08 pg/mL for myoglobin, 0.452 ppm for toluene, and quick response/recovery rates have been produced using biopolymers via a variety of fabrication strategies, such as electrospinning, hydrogel crosslinking, molecular imprinting, and in situ nanoparticle assembly. GO, carbon dots, quantum dots, CNTs, and fluorophores were used to functionalize chitosan, cellulose, PVA, PCL, PLA, and PHA-based composites to obtain excellent electromechanical coupling, fluorescence switching, and retention of ions. Bio-actuators made of biopolymers in conjunction with ionic liquids, nanoparticles, or photo-responsive moieties (e.g., azobenzene, anthracene) and engineered through electrospinning, freeze-drying, or bilayer templating have been used to provide selective responsive electromechanical outputs, including reversible bending, generation of force, and shape programming. Bio-actuators demonstrated remarkable performance, with actuation at low voltages (1–10 V), sunlight, humidity, and vapor stimuli, achieving tip deflections greater than 49 mm, strains exceeding 150%, and the capacity to lift objects multiple times their weight. The application of biopolymers in healthcare emphasizes versatile performance, including chitosan-modified (PCLP-CUR) carriers, which have demonstrated improved tumor-specific delivery, pH-responsive release, and the ability to avoid macrophages. Similarly, biopolymer blends, such as chitosan/Bletilla striata polysaccharide and carrageenan/PVA, exhibited superior mechanical properties and antibacterial activities, with potential applications in wound healing and dental tissue engineering. Interestingly, electrospun nanofibrous membranes incorporating antimicrobial materials, such as eucalyptus oil, cinnamon extract, or silver nanoparticles, demonstrated sustained release kinetics, cytocompatibility, and enhanced wound healing in both non-living and living models. This combination of desirable hemocompatibility, low cytotoxicity, and high cell adhesion properties has allowed biopolymers to become versatile and sustainable materials for future healthcare devices and therapies.

However, several key challenges must be addressed before biodegradable polymers can be widely adopted in the industry, including scalability, purification process, mechanical integrity, controlled and desired degradation, recyclability, integration into high-performance systems, and regulatory standardization. Not all biopolymers biodegrade immediately; it is a combination of chemical composition, processing, and disposal conditions. A comprehensive understanding of the biodegradation mechanism is essential to optimize the routes they are handled at the end of life. Moreover, Cradle-to-Cradle Life Cycle Assessment (LCA) studies explain the sustainability claims due to their potential to have hidden environmental expenses in feedstock growth, the utilization of water, and energy-consuming extraction methods. Integrating LCA with performance provides a holistic insight into their applications over conventional petroleum-based polymers. Therefore, the field is at a point between innovative breakthroughs and practical implementation, requiring interdisciplinary efforts to enhance performance while ensuring biodegradability, compliance with LCA standards, and circularity. A circular-economy approach to plastics involves designing materials to circulate in low-waste, closed loops rather than relying on alternative sources of carbon [142]. These challenges can be addressed by switching to renewable feedstocks. Still, the process should also include policies and design rules that extend product lifespans and enable recovery at the end of life. This approach is supported by EU initiatives in the Green Deal and the Circular Economy Action Plan, which emphasize durability, repairability, reuse, high-quality recycling, energy and resource efficiency, and verifiable low-carbon performance throughout the product life cycle [143,144]. In this context, biopolymers have renewable carbon and, when designed appropriately, end-of-life solutions compatible with local infrastructure like industrial composting, anaerobic digestion, or recycling [145]. In biodegradable composites, materials like CNT, graphene, and polyaniline can form electrical pathways at very low concentrations close to the percolation threshold, reducing the amount of material needed and lowering risks [146,147]. Sustainability can also be enhanced by immobilizing or covalently attaching fillers to the matrix, so they are not shed during use or at EoL, using benign dopants in polyaniline, eliminating persistent fluorinated agents, and replacing persistent fluorinated aids with biobased and mineral dispersion aids. CNTs and graphene sheets may be compatible with compostable or soil-biodegradable systems at realistic concentrations, provided that the final products are tested for migration and ecotoxicity, and that release does not exceed safe limits during simulated composting, anaerobic digestion, and abrasion. This approach is similar to the use of magnetic or ceramic nanofillers, and ionic liquids should also be considered, as long as their chemistries exhibit low acute toxicity and they remain within the matrix during degradation [148]. This strategy enables biopolymer composites to deliver conductivity and mechanical performance while remaining compatible with end-of-life routes that are safe and suited to specific infrastructure. When addressed comprehensively, biopolymers have the potential to serve as fundamental materials for a future generation of smart, sustainable, and intelligent energy storage systems.

## Figures and Tables

**Table 2:** Comparative study of biodegradable composites for electronic applications and their key characteristics.

Biopolymers	Application	Processing	Key Findings	Characteristics	Ref.
Starch/metal halide	Biopolymer gel electrolyte	Extruder	Electrical conductivity increased to 10^−6^ S/cm	While materials are moisture-sensitive, water content does not significantly affect the conductance of doped films The mechanical characteristics show that the starch is plasticized, and the ion-conducting material is durable and easy to manage	[49]
Starch/inorganic salt	Flexible electronic sensor	Thermogelation	The healing lasts 2–3 s without special environment, with 98% efficiency	The composite hydrogel demonstrates outstanding performance in circuit repair A novel strategy uses natural hydrogen-bonded materials as structural frameworks for electrode materials in flexible electronic devices.	[50]
Starch/montmorillonite/1-allyl-3-methylimidazolium chloride (IL)	Ion-conducting biopolymer nanocomposite	Solution Casting	Ionic conductivity increased to 10^−3^ S/cm	Hydrophilic ILs, like those with high chloride ions, form strong hydrogen bonds with starch.	[51]
Starch/1-ethyl-3-methylimidazolium acetate (IL)	Electroconductive films	Compression moulding	Ionic conductivity increased to 0.0118 S/cm with low processing temperature (55 °C) and a higher relative humidity (75%)	Adding IL reduced the thermal stability of starch-based films by 30 K, regardless of formulation or processing.	[52]
Chitosan/PVA/silica	Proton exchange membrane	Solution casting	Proton conductivity is 5.08 × 10^−4^ S/cm at 100 °C The addition of silica also increases the water content to 55.7%,	Silica filler loading (0.5–10 wt.%) varies to study its effect on membranes’ temperature-dependent proton conductivity.	[54]
Chitosan/PVA/ SSA	Proton conducting membranes	Solution casting	Proton conductivity showed a maximum of 3.9 × 10^−3^ S/cm at 20% loading of the nanoparticles.	The membranes displayed strong resistance to hydrolysis, heat, and oxidation.	[55]
Chitosan/silicotungstic acid	Proton-conducting membranes for fuel cells	Ionotropic gelation	Proton conductivity of the membrane at 25 °C in a hydrogen fuel cell achieves a maximum power density of 268 mW/cm^2^ and i_0_ of 12 µA/cm^2^ with 0.5 mg/cm^2^ Pt loading.	Inside the membrane, the heteropolyacid’s Keggin structure remains intact even after fabrication The polarization curves analysis showed good O_2_ reduction activity, confirmed by the kinetic parameters from the best EIS spectra fit.	[56]
Cellulose nanocrystal/protic ionic liquid(PIL)	Biopolymer electrolyte membrane for a Fuel cell	Solution casing	Proton conductivity of 10^−4^–10^−3^ S/cm at 160–120 °C	Biopolymer’s apparent nanoscale dimensions reveal how CNCs’ defects, twisting, and aggregation depend on the PIL The fuel cell setup shows membranes responding to H_2_ inlet, producing an electrical current The temperature range for optimal performance of the membrane exceeds the target temperature for future non-humidified fuel cell devices	[57]
Carboxylated chitosan/acrylic acid/ N, N′-methylene bisacrylamide	Supercapacitor	Grafting copolymerization	Shows high ionic conductivity of 7.82 × 10^−2^ S/cm and a high electrolyte uptake of 524% The energy and power density are notably high, measured at 4.39 Wh/kg and 224.99 W/kg, respectively.	These membranes can be used in any condition, whether acid, alkali, or neutral, for supercapacitors.	[58]
Starch/CaCl_2_/glycerol	Strain sensitive batteries and self powered sensors	Solution mixing	1.5371 kPa^−1^ sensitivity of self-powered sensors After 1000 compression cycles at 20% strain, the battery’s output current stayed responsive, demonstrating strong pressure sensitivity stability.	This starch/glycerol hydrogel is highly stretchable with elongation at break 208 ± 24% and has a tensile sensitivity (GF: 1.35) It withstands 1000 cycles of 100% stretch or 40% compression with minimal sensitivity change.	[62]
PLA/PEG	Separator for supercapacitors	Phase inversion and radio frequency air plasma method	Ionic conductivities were 1.1 × 10^−1^ S/cm and 0.6 × 10^−2^ S/cm in 1 M H_2_SO_4_ and 1 M N_a_2SO_4_, respectively The system showed lowest internal resistance of 1.9 Ω and solution resistance	PEG removal resulted in highly porous structure RF-plasma treatment produced a highly hydrophilic surface	[63]
Chitosan/hydroxyapatite	Thermal insulation	Crosslinking and freeze drying	Lower thermal conductivity up to 37.43 mW/mK	Excellent mechanical strength of 0.82–2.37 MPa with specific modulus up to 129.20 kN·m/kg	[71]

**Table 3:** Comparative study of biodegradable composites for sensor applications.

Sensor Type	Biopolymer Matrix	Analyte Detected	Sensitivity	Response Time	Durability	Ref.
Electrochemical sensing	Chitosan/PVA	Ethylene	~80% at 200 ppm	<30 min (180 ppm)	--	[75]
Fluorescence-based sensor	Chitosan	Ferrous ion (Fe^2+^)	89.4%; LOD-0.55 µM	20 min for maximum equilibrium adsorption 223.5 g/mg	>5 cycles	[76]
Fluorescence-based sensor	PCL	Ferric ion (Fe^3+^)	LOD 2.94 nM	1 min		[77]
Strain sensor	PVA/Lignin	Strain	For the gauge factor, 137.3 and 2746.4, corresponding to strain ranges of 0–160% and 160–240%, respectively.	Fast	>10,000 cycles	[79]
Pressure sensor	PCL/TPU	Pressure/acoustic	Within 0–34 kPa, sensitivities of the planar and grid sensors were 1.80 kPa^−1^ and 2.24 kPa^−1^; in the 35–75 kPa range, they were 1.03 kPa^−1^ and 1.27 kPa^−1^.	Dynamic response, 0–0.5 Hz is 55 dB	dynamic cyclic stability ~1200 s	[85]
Electrochemical sensor	PL/PEG	Ascorbic acid and Trolox	LOD 8.12 µM for ascorbic acid and 3.53 µM for Trolox	--	--	[93]
Gas sensor	Gelatin/agar/alginate	Volatile organic compounds	96%	35 s VOC headspace exposure, 100 s recovery; robustness test: 30 s exposure/30 s recovery	100 cycles	[95]
Human movement monitoring sensor	Starch/PVA	Strain	Gauge factor is 0.74	--	200 cycles	[97]

## Data Availability

No new data were created or analyzed in this study. Data sharing is not applicable to this article.

## Abbreviations

PHAs	Polyhydroxyalkanoates
PLA	Polylactic acid
PGA	Polyglycolic acid
PCL	Polycaprolactone
PBS	Poly (butylene succinate)
PLGA	Poly (lactic-co-glycolic acid)
MWCNT	Multi-walled carbon nanotubes
PVA	Polyvinyl alcohol
IEC	Ion-exchange capacity
IPN	Interpenetrating network
CS or CH or CHT or CSBHD	Chitosan
SSA	Sulfosuccinic acid
PEM	Proton-exchange membrane
CNC	Cellulose nanocrystals
PIL	Protic ionic liquid
RF	Radio Frequency
SEM	Scanning electron microscope
BioPEM	Biopolymer electrolyte membranes
HPMC	Hydroxypropyl methylcellulose
EDLC	Electric Double-Layer Capacitors
RGO	Reduced graphene oxide
UT	Ultrasonic transducer
FASC	Flexible all-solid-state supercapacitor
SA	Sodium alginate
GO	Graphene oxide
CV	Cyclic voltammetry
LIF	Laser-induced fluorescence
FPN	4,4′-fluoresceinoxy bisphthalonitrile
LOD	Limit of detection
NCD	N-doped carbon dots
AIEE	Aggregation-induced enhanced emission
MIP	Imprinted chitosan
AA	Ascorbic acid
PEG	Polyethylene glycol
PET	Photo-induced electron transfer
NBU-CD	Fluorescent nanofiber membrane
U-CD	Xylan-derived carbon dots
HEC	Hydroxy ethyl cellulose
QD	Quantum dots
CQD	Carbon quantum dots
BTX	Benzene, Toluene, and Xylene
LDH	Layered double hydroxide
TPU	Polyurethane
AuNC	Gold nanoclusters
AMP	Adenosine 5′-monophosphate
PEDOT:PSS	Poly(3,4-ethylenedioxythiophene) polystyrene sulfonate
ECG	Electrocardiogram
CNT	Carbon nanotube
VOC	Volatile organic compound
Ra	Relative response
GF	Gauge factor
Azo	Azobenzene
CBC	Carboxylated bacterial cellulose
IL	Ionic liquid
MFC	Microfibrillated cellulose
CMCS	Carboxymethyl chitosan
TA	Tannic acid
CMC	Carboxymethyl cellulose
SCIA	Sodium alginate-carboxymethyl cellulose ion actuator
9-AN	9-anthracenecarboxylic acid
THBP	2,2′,4,4′-tetrahydroxybenzophenone
BPBE	Biopolymer blend electrolyte membranes
κ-Car	κ-Carrageenan
DNH	Double-network hydrogel
AMPS	2-Acrylamido-2-methylpropane sulfonic acid
FBAM	Flexible biomimetic artificial muscle
SIO	Silk fibroin inverse opal
PDMS	Polydimethylsiloxane
CTE	Coefficient of thermal expansion
PVDF	Polyvinylidene fluoride
CUR	Curcumin
PCLP-CUR	CUR-loaded platelet membrane bioinspired chitosan liposome
BSP	Bletilla striata polysaccharide
DPPH	1, 1-diphenyl-2-picrylhydrazyl
AgNP	Silver nanoparticles
APTES	3-aminopropyltriethoxysilane
PHB	Polyhydroxybutyrate
DMAEMA	Dimethylaminoethyl methacrylate
CNF	Chitin nanofibrils
TEER	Transendothelial electrical resistance
SL	Soda lignin
ASL	Acetylated soda lignin
HMK	High molecular weight keratin
ALP	Alkaline phosphatase
MFC-IL	Microfibrillated cellulose-ionic liquid
PHBV	Poly(3-hydroxybutyric acid-co-3-hydroxyvaleric acid)
